# Interrogation of the Intermolecular Forces That Drive Bulk Properties of Molecular Crystals with Terahertz Spectroscopy and Density Functional Theory

**DOI:** 10.1021/acs.cgd.5c00007

**Published:** 2025-05-23

**Authors:** William B. Stoll, Peter A. Banks, Steven G. Dannenberg, Rory Waterman, Luca Catalano, Michael T. Ruggiero

**Affiliations:** †Department of Chemistry, ††Department of Chemical Engineering, 6927 University of Rochester, 120 Trustee Road, Rochester, New York 14627, United States; ‡ Laboratory for Chemistry of Novel Materials, 54521 University of Mons, Place du Parc 20, B-7000 Mons, Belgium; § Department of Chemistry, 2092 University of Vermont, 82 University Place, Burlington, Vermont 05405, United States; ∥ Dynamic Molecular Materials Laboratory, Dipartimento di Scienze della Vita, Universitá degli Studi di Modena e Reggio Emilia, Via Campi 103, 41125 Modena, Italy

## Abstract

Identifying and characterizing intermolecular forces in the condensed phase is crucial for understanding both micro- and macroscopic properties of solids; ranging from solid-state reactivity to thermal expansion. Insight into these interactions enables a holistic comprehension of bulk properties, and thus understanding them has direct implications for supramolecular design. However, even modest changes to intermolecular interactions can create unpredictable changes to solid-state structures and dynamics. For example, copper­(II) acetylacetonate (Cu­(C_5_H_7_O_2_)_2_) and copper­(II) hexafluoroacetylacetonate (Cu­(C_5_HF_6_O_2_)_2_) exhibit similar molecular conformations, yet differences between the methyl and trifluoromethyl groups produce distinct sets of intermolecular forces in the condensed phase. Ultimately, these differences produce unique molecular arrangements in the solid state, with corresponding differences in material properties between the two crystals. In this work, terahertz spectroscopy is used to measure low-frequency vibrational dynamics, which, by extension, provide detailed insight into the underlying intermolecular forces that exist in each system. The experimental data is coupled to theoretical quantum mechanical simulations to precisely quantify the interplay between various energetic effects, and these results highlight the delicate balance that is struck between electronic and dispersive interactions that underpin the structural and related differences between the two systems.

## Graphical abstract

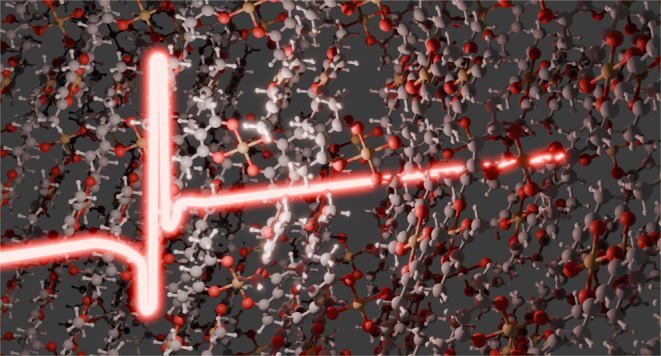

## 1 Introduction

Noncovalent interactions, while typically weak, underpin many processes that dictate the macroscopic properties of condensed phase materials, including thermal contraction and expansion,^1–3^ mechanical effects,^4–6^ reactivity,^7^ and gas adsorption in porous materials.^8–10^ While noncovalent forces are important for all phases of matter, they are particularly impactful for solid-state properties. Despite typically comprising less than ca. 1% of the total energy of molecular crystals, noncovalent interactions are ultimately responsible for the formation of crystalline solids, as well as many of the resulting macroscopic properties.^11–13^ This is one reason that molecular crystals exhibit a rich polymorphic landscape, which arises from the interplay between conformational energy and noncovalent interactions, specifically intermolecular forces (IMFs).^14–16^ This occurs due to the balance that can be struck between conformational strain and stabilizing IMFs.^17,18^ A deep understanding of IMFs is required to design, manipulate, and tune preferable macroscopic properties of materials.^19^ Methodologies commonly used to investigate IMFs involve a comparison of systems that include a structural alteration, i.e., functional group exchanges,^20,21^ polymorphic crystals,^22,23^ isostructures,^24^ or *cis*/*trans* isomers.^25,26^ However, IMFs that drive supramolecular properties are often deeply entangled, making them difficult to quantify individually.^27^ While traditional mid-infrared (mid-IR) spectroscopic methods have been used to probe IMFs, such tools rely on extracting information about noncovalent interactions indirectly–for example, by observing the change in a covalent bond strength based on IMFs.^28,29^ Structural methods, on the other hand, also do not provide quantitative insight into IMFs, again requiring that their influence be characterized empirically based on quantities like bond length.^30,31^

These limitations have necessitated the exploration of techniques better suited for the study of weak, noncovalent, interactions in molecular crystals. Over the past decade, terahertz time-domain spectroscopy (THz-TDS) has become an established tool for investigating IMFs in the condensed phase. This is because the motions probed using low-frequency (terahertz) vibrational spectroscopy (0.1–10 THz, 3–333 cm^–1^, λ = 0.3 mm to 30 μm) often involve large-amplitude displacements of either entire molecules or large-portions of molecules (torsions).^32–35^ Because the vibrational coordinates involved in terahertz dynamics are highly influenced by weak and noncovalent interactions, THz-TDS is an ideal tool for directly (rather than indirectly) characterizing the intermolecular coordinate.^36–41^

The strong dependence of low-frequency vibrational dynamics on long-range forces means that each unique three-dimensional arrangement of atoms/molecules produces a unique terahertz spectrum. Thus, unlike the well-known tables of functional group specific transitions that exist in the mid-IR, no corresponding standardized data exists for the terahertz region. Because of this, while it is common to analyze mid-IR spectra without the use of theoretical tools (as the spectra can be readily assigned), experimental terahertz spectra on their own are difficult to analyze in isolation. This has necessitated the development of theoretical tools to aid in the assignment and interpretation of experimental THz-TDS data, in many cases based on solid-state density functional theory (ss-DFT).^32,34^ Recent advancements in ss-DFT have proved useful in quantifying these low-energy forces, but depend heavily on the accuracy of the modeled potential energy surface (PES).^42^ This model is dependent on user-selected parameters (e.g., functional, basis set, reciprocal space sampling, and so on), and thus different combinations of these parameters produce unique hyper-surfaces where minor inaccuracies can have a profound effect on calculated values.^43–45^ To confidently model materials, the simulations must be validated through comparison of experimental data. Due to the sensitivity of THz-TDS to weak interactions, a highly accurate computational model of a system will produce a terahertz spectrum in close agreement with experiment–implying a good representation of the weak noncovalent forces found within the system.^18,46^ This agreement represents a powerful combined data set, and enables a confident investigation and quantification of IMFs in molecular crystals. Consequently, precise agreement between experimental terahertz spectra and theoretically calculated spectra not only validates computational predictions, but also ensures confidence in their accuracy. This rigorous validation step transforms theoretical calculations from mere numerical predictions into reliable and meaningful representations of the underlying intermolecular forces.

In this work, two systems were chosen that exhibit very similar conformational geometries, but differ greatly in their macroscopic properties, such as their three-dimensional lattices and associated mechanical responses. Copper­(II) acetylacetonate (Cu­(acac)_2_), is a coordination complex that crystallizes in monoclinic (*P*2_1_/*c*) unit cell, and displays π-stacking in the [010] direction with a herringbone pattern in the (100) and (1̅00) planes. The π-stacking motif of this structure allows for the dissipation of external mechanical stress into the intermolecular interactions, resulting in a crystal that can bend elastically.^47,48^ Copper­(II) hexafluoroacetylacetonate (Cu­(hfac)_2_), while exhibiting a similar molecular conformation, crystallizes in a triclinic (*P*1̅) structure that forms sheets in the (11̅0) plane, but to the best of our knowledge has not been reported to exhibit elastic properties like those of Cu­(acac)_2_. A hydrogen-to-fluorine substitution, while subtle,^49^ sufficiently alters the weak IMFs and associated dynamics, giving rise to contrasting bulk properties. To understand the reciprocity of the electronic and dispersive interactions that govern the bulk properties of each system, we employ THz-TDS and ss-DFT to directly probe these weak interactions, providing fundamental insight into structure/function properties of molecular crystals. Leveraging this insight provides another opportunity for the design of molecular (dynamic) crystals with exotic properties.

## 2 Methods

Cu­(acac)_2_ (99%, Sigma-Aldrich) and Cu­(hfac)_2_ (99%, Sigma-Aldrich) were obtained commercially. While Cu­(acac)_2_ was used as-received, Cu­(hfac)_2_ is hygroscopic and the original sample is primarily Cu­(hfac)_2_·3H_2_O, which was confirmed by powder X-ray diffraction (see Supporting Information (SI)). Thus, to generate anhydrous Cu­(hfac)_2_, the samples were first dried in a desiccator. Samples were then suspended in a poly­(tetrafluoroethylene) (PTFE) matrix for spectroscopic measurements by mixing with PTFE (to a 3% w/w concentration) and grinding to homogenize the mixture and to reduce particle size to minimize scattering.^50^ The resulting powder was pressed into pellets using a 13 mm diameter die and hydraulic press (Specac, 225 MPa), yielding approximately 3 mm tall free-standing pellets. The samples were then placed into a sample-in-vacuum cryostat (Lakeshore Cryotronics), and the entire system was vacuumed overnight to ensure that Cu­(hfac)_2_ was in its anhydrous form prior to performing the THz-TDS experiments.

THz-TDS was utilized to probe low-frequency vibrational motions with a commercial spectrometer (Teraflash, Toptica Photonics). The free-space optical setup utilized a fiber-optic emitter and receiver, paired with four off-axis parabolic mirrors in a U-shape configuration to focus and collimate the terahertz beam.^51^ The setup was enclosed and continuously purged with dry nitrogen gas to eliminate absorption from atmospheric water vapor. Three measurements were acquired for both the sample and blank pellet at cryogenic (LN_2_) temperature. Each final spectrum represents the average of 20 thousand waveforms per measurement, across the three repeated sample and blank pairs. These waveforms were zero padded by adding 8000 points to both ends (16000 total points) and subjected to a Hann function. The time-domain data were Fourier transformed to yield a frequency-domain power spectra. Each sample was divided by a respective reference PTFE pellet spectrum to yield a frequency-domain spectrum with a bandwidth of 20–120 cm^–1^. The spectra presented are a result of three averaged absorption spectra.

Periodic ss-DFT calculations were performed with the Crystal23 software package.^52^ Single-crystal X-ray diffraction (XRD) structures of Cu­(acac)_2_ ^53^ and Cu­(hfac)_2_ ^54^ were obtained from the Cambridge Crystallographic Data Centre (CCDC), and were used as initial structures for all calculations. Geometry optimizations were performed first, allowing a full relaxation of lattice parameters and atomic positions, ensuring all atomic nuclei were located at a minimum on the PES, with a convergence criterion of Δ*E* < 10^–8^ Hartree. The generalized gradient approximation PBE functional,^55^ including Grimme’s D3 dispersion correction (PBE-D),^56,57^ was used along with an atom-centered triple-ζ basis set (POB-TZVP)^58^ for all calculations.

Electronic energies were determined with the unrestricted-spin self-consistent field (SCF) method.^59^ The calculation of vibrational modes was performed numerically at the Γ-point within the harmonic approximation,^60,61^ and intensities were calculated through the dipole moment derivatives using the Berry phase method.^62^ Vibrational spectra were plotted using a Lorentzian function, with peak widths determined empirically based on the experimental spectra. Cu­(acac)_2_ and Cu­(hfac)_2_ were fit with a full width half max of 8 and 5 cm^–1^, respectively. Mulliken charges were calculated using the Properties postprocessing module within the Crystal23 code. Basis set superposition error was quantified using the counterpoise correction method.^63^ Thermoelastic constants were calculated using the quasi-harmonic approximation.^64–69^

## 3 Results and Discussion

### 3.1 Structural Analysis by X-ray Diffraction and Density Functional Theory

#### 3.1.1 Cu­(acac)_2_

Cu­(acac)_2_ crystallizes in the monoclinic *P*2_1_/*c* space group (Figure 1), containing half a molecule in the symmetry-independent unit (*Z*′ = 0.5) and two total molecules in the unit cell (*Z* = 2). X-ray diffraction (*T* = 100 K) reveals lattice dimensions of *a* = 11.282 Å, *b* = 4.625 Å, *c* = 14.936 Å, and a β angle of 136.648° (CCDC deposition: 281026).^53^ A complete structural optimization using ss-DFT predicted lattice dimensions of *a* = 10.965 Å, *b* = 4.543 Å, *c* = 14.355 Å, and a β angle of 135.950°, with a density of the unit cell of 1.74 g/cm^3^. It should be noted that there is a slight overall compression of the Cu­(acac)_2_ unit cell after optimization, with the cell volume decreasing from 535.001 to 497.246 Å^3^, representing an average decrease of 2.17% compared to the experimental 100 K XRD structure. One noteworthy consequence of the Cu­(acac)_2_ structure is its ability to bend elastically, hypothesized to be due to the nature of the crystal structure and associated molecular dynamics.^47^ To provide further confidence in the accuracy of the utilized theoretical model, the elasticity of the system was also modeled. Worthy et al. experimentally determined Young’s modulus along the [101] and [101̅] crystallographic directions as 11.3–13.8 and 4.8–6.9 GPa, respectively,^70^ while the thermoelastic calculations performed here found a Young’s modulus of 14.4 and 3.5 GPa for the [101] and [101̅] directions, respectively, in excellent agreement with the experimental and previously reported theoretical data.^71^

**Figure 1:**
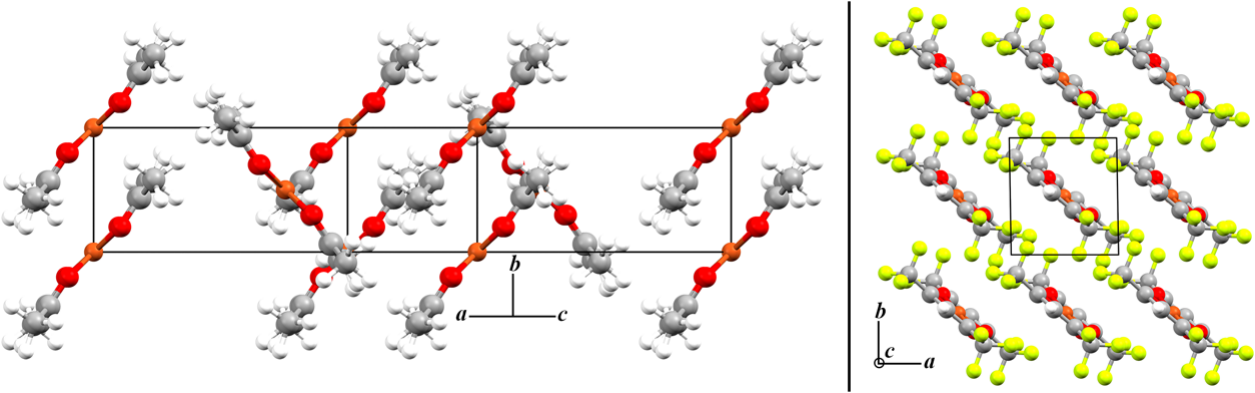
X-ray diffraction structures of Cu­(acac)_2_ (left) and Cu­(hfac)_2_ (right). White, gray, red, orange, green atoms are hydrogen, carbon, oxygen, copper, and fluorine, respectively.

Molecules of Cu­(acac)_2_ stack along the crystallographic *b*-axis in an eclipsed conformation, with a herringbone pattern along the (100) and (1̅00) planes. Intermolecular interactions were first identified upon visual inspection of the crystal structure, in which three unique close contacts arise (Figure 2). Dimer pair A consists of π-stacked pseudosix-membered rings in a “sandwich” conformation. The average distance between atoms in the eclipsed ring is 3.101 Å, with a simulated average of 3.004 Å. Dimer pair B arises from the tip-to-tail nature of the herringbone structure, which involves dispersive forces between the hydrocarbon portions of each molecule. Dimer pair C involves both dispersive forces and a weak hydrogen bond; the X-ray structure exhibits a 3.016 Å C–H···O hydrogen bond (C···O distance), which was accurately simulated with a predicted C–H···O hydrogen bond distance of 3.090 Å.

**Figure 2:**
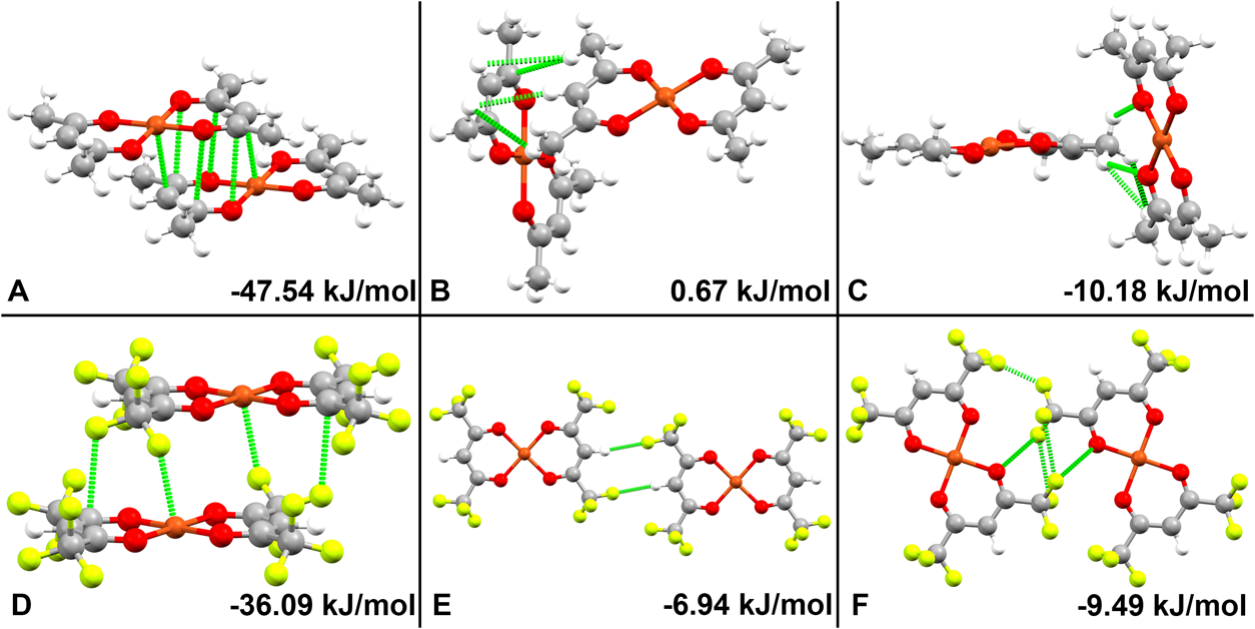
Dimer pairs extracted computationally from the crystal structures of Cu­(acac)_2_ (top, A–C) and Cu­(hfac)_2_ (bottom, D–F). Dimer pair labels and dimerization energy are presented in the bottom left and right of each panel, respectively. Dotted green lines express close contacts (distances closer than the sum of the van der Waal radii).

#### 3.1.2 Cu­(hfac)_2_

Cu­(hfac)_2_ crystallizes in the triclinic *P*1̅ space group (Figure 1), also containing half a molecule in the symmetry-independent unit (*Z*′ = 0.5) with one molecule in the unit cell (*Z* = 1). Experimental single-crystal X-ray diffraction (*T* = 100 K) reveals lattice dimensions of *a* = 5.428 Å, *b* = 5.849 Å, *c* = 11.516 Å, α = 81.470°, β = 74.573°, and a γ = 86.960° (CCDC deposition: 201577).^54^ A complete geometry optimization with ss-DFT predicted lattice dimensions of *a* = 5.394 Å, *b* = 5.708 Å, *c* = 11.613 Å, α = 81.634°, β = 74.391°, and a γ = 86.838°, with a density of the unit cell of 2.324 g/cm^3^. It can be noted that after geometry optimization, there was a slight overall compression of the Cu­(hfac)_2_ unit cell volume decreasing from 348.510 to 340.680 Å^3^.

Three unique close contacts arise from molecules of Cu­(hfac)_2_ layering in sheets along the (11̅0) plane, displayed in Figure 2. Dimer pair D exhibits a stacked structure which gives rise to an electrostatic fluorine–copper interaction (SI Figure 1), closely resembling Jahn–Teller distortion with experimental 180° F···Cu···F angle and 2.709 Å F···Cu distances (simulations found a 180° angle and 2.649 Å bond distances). Dimer pair E is the tip-to-tail interaction of the sheet and involves a repulsive electrostatic interaction as well as attractive dispersive interactions. In this system, the intermolecular H···F bond lengths are 2.540 Å (2.450 Å simulated) with a C–F···H angle of 176.660° (175.750° simulated), which would be electrostatically repulsive. Dimer pair F includes dispersive forces as well, in which two parallel C–F bonds have an F···F spacing of 3.270 Å (3.210 Å simulated).

### 3.2 Vibrational Analysis

Vibrational spectroscopy is a powerful tool to directly probe interatomic and intermolecular forces, as the normal-mode frequencies are dependent on the second derivative (i.e., force constant) of the vibrational potential energy. In this study, THz-TDS is used in two respects; as a validation tool for ss-DFT and to understand how the lattice dynamics are related to both the forces and macroscopic properties.

THz-TDS is a highly sensitive probe of crystalline structure and dynamics, both of which are governed by long-range IMFs. Therefore, an accurate computational model of these IMFs should reproduce the low-frequency vibrational spectra with close agreement to experiment.^43,72^ The experimental and theoretical THz-TDS spectra for Cu­(acac)_2_ and Cu­(hfac)_2_ are shown in Figure 3, and display good overall agreement in both frequency and intensity, supporting the accuracy of the calculated intermolecular potential energy surfaces and charge distributions. However, there are some discrepancies that should be noted. The spectrum of Cu­(hfac)_2_ contains a few subtle absorption features that are not fully captured by the theoretical simulation—these might indicate a small contamination of the sample from residual hydrate composition (see SI for Cu­(hfac)_2_ hydrate THz-TDS spectra). Closer inspection reveals that the simulated spectra are systematically blue-shifted relative to the experimental data. This effect is well-known in DFT-based harmonic frequency calculations, where it is common to apply empirical scaling factors to approximate anharmonic corrections and basis set limitations.^73–75^ In the present study, a uniform scaling factor of 0.9 brings the simulated spectra into excellent agreement with experiment (see Supporting Information). Several physical origins can contribute to this blue shift. First, the simulations are performed at 0 K, while the experiments were conducted at 78 K. The absence of thermal expansion in the fully unconstrained simulations leads to contracted unit cell volumes, which increase intermolecular interaction strengths and elevate vibrational frequencies. Additionally, harmonic approximations inherently neglect anharmonic contributions that tend to red-shift vibrational frequencies, particularly at low energies.^76,77^ Finally, the Cu­(acac)_2_ experimental spectrum contains a rising background signal, likely caused by scattering of the terahertz radiation by either the crystalline particles or porous air voids in the tablet.^78^ This leads to a large apparent disagreement with the theoretical intensities, as the simulation does not take these macroscopic effects into account. However, adding the experimental background signal to the theoretical spectrum results in much better agreement with experiment (see Supporting Information). Despite these systematic effects, we have chosen to report the unscaled harmonic spectra throughout this work in order to preserve consistency with our subsequent analyses of vibrational energetics and mode-resolved force profiles (vide infra). The close correspondence between relative spectral features supports the reliability of the theoretical description in capturing the essential structural dynamics of both materials.

**Figure 3:**
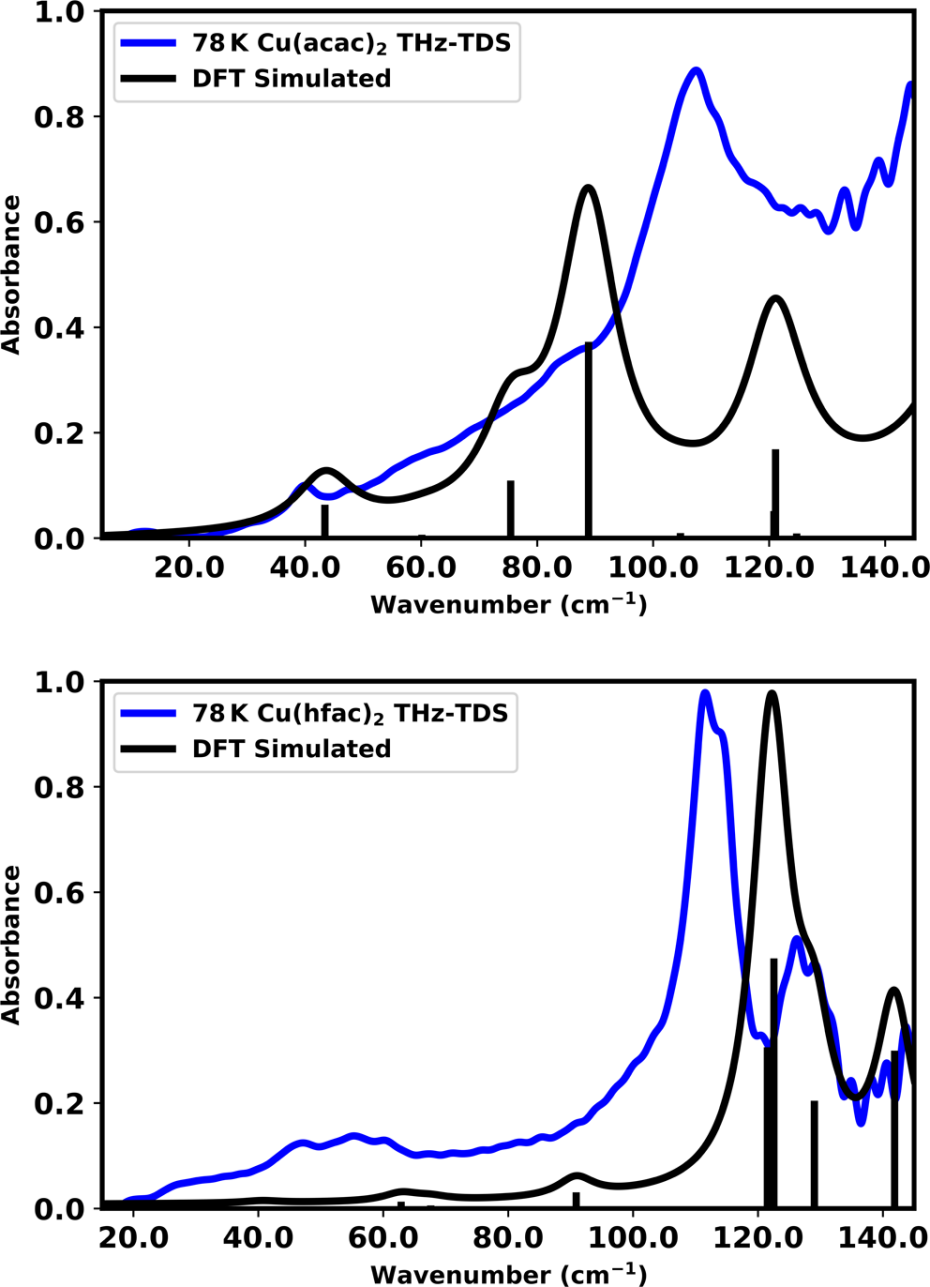
Experimental 78 K THz-TDS spectra (blue) and ss-DFT predicted spectra (black) of Cu­(acac)_2_ (top) and Cu­(hfac)_2_ (bottom).

Intermolecular vibrations were visualized using the eigenvector displacements (produced by the ss-DFT calculations) to further understand the dynamics and forces present in each system. Low-frequency vibrational modes arise directly from the curvature of the intermolecular PES,^79^ and therefore, a visualization of these modes provides information about the forces that drive the motions.^33,36^ Many of the modes involve conformational changes or translations of the molecules. The eigenvector displacements of modes 120.81, 121.09 cm^–1^ for Cu­(acac)_2_ and 129.02 cm^–1^ for Cu­(hfac)_2_ are illustrated in Figure 4 (animations and frequencies of all terahertz active modes are provided in the Supporting Information). The 120.81 cm^–1^ mode (Figure 4I) involves an in-plane scissoring motion of the entire molecule, which is facilitated by dispersive forces between the perpendicular pairs of molecules. The 121.09 cm^–1^ mode (Figure 4II) involves an out-of-plane scissoring of the entire molecule, in which the copper and oxygen molecules displace toward adjacent ring, arising from the π–π interaction. The 129.02 cm^–1^ mode (Figure 4III) of Cu­(hfac)_2_ involves an out-of-plane conformational distortion of the copper ion as well as a bend of the trifluoromethyl, effectively producing an intermolecular stretching between F···Cu···F. Overall, visual inspection of the three-dimensional structure and the eigenvector displacements of low-frequency modes provides a useful starting point in the analysis of IMFs, but to gain further insight, a precise energetic analysis was carried out.

**Figure 4:**
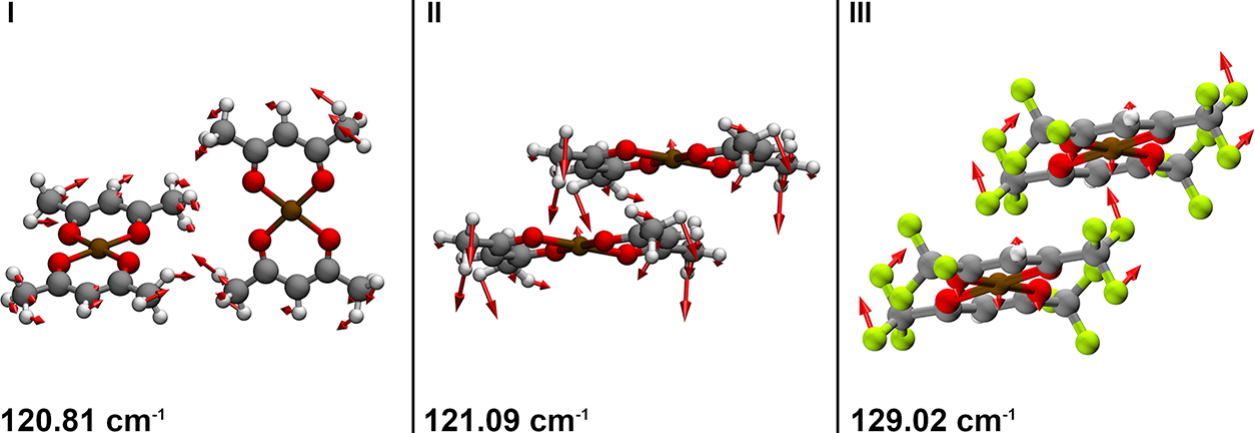
Phonon modes in Cu­(acac)_2_ (I, II) and Cu­(hfac)_2_ (III) where the red arrows are representative of the displacement of the atoms along the vibrational coordinate.

### 3.3 Energetic Analysis by Density Functional Theory

A strategy we employed to investigate IMFs involved the calculation of dimerization energies of computationally extracted pairs of molecules from the crystal lattice. The binding energies of the six dimer pairs presented in Figure 2 were determined using the equation

$$E_{\mathrm{binding}}=E_{\mathrm{dimer}}-\left(2\times E_{\mathrm{monomer}}\right)-\overset{\mathrm{BSSE}\;\mathrm{correction}}{\overset{︷}{\left(\left(E_{\mathrm{ghost},A}-E_{\mathrm{monomer},B}\right)+\left(E_{\mathrm{ghost},B}-E_{\mathrm{monomer},A}\right)\right)}}$$

where *E*_binding_ is the total binding energy (electronic plus dispersion) arising from dimerization, *E*_dimer_ and *E*_monomer_ are the total energies of the dimer and isolated monomer, respectively, and *E*_ghost_ is the energy of the monomer calculated in the presence of ghost atoms. A ghost atom is defined as a position where basis functions are present, but no electrons or nuclei are assigned. These are used to evaluate and correct for basis set superposition error (BSSE), which arises when the basis functions of neighboring molecules artificially stabilize the calculated energy. Importantly, because dispersion energies are determined separately from the electronic energy, it is straightforward to partition calculated values into these two energetic origins. The contributions of electronic and dispersive interactions to the total dimerization energy are displayed in Table 1.

**Table 1:** Binding Energies of Dimer Pairs, with Contributions from Dispersive and Electrostatic Interactions^a^

system	dimer pair	dimerization energy	dispersion contribution	electronic contribution
Cu(acac)_2_	A	–47.54	–60.19	12.66
	B	0.67	–17.78	18.45
	C	–10.18	–9.95	–0.22
Cu(hfac)_2_	D	–36.09	–37.83	1.74
	E	–6.94	–7.03	0.09
	F	–9.49	–18.80	9.31

a All energies are reported in kJ mol^–1^.

It is important to mention that the DFT-D3 approach is a widely used and computationally efficient method to estimate dispersion forces in molecular crystals,^80,81^ but is not without limitations. In particular, the method relies on parameterized atom-pairwise corrections that can, in some cases, lead to overbinding due to partial double-counting of dispersion effects already captured in the base functional.^82,83^ Despite these known issues, we emphasize that in the systems studied here, the dispersive interactions dominate the total dimerization energy by a significant margin (Table 1). As such, modest inaccuracies in the absolute dispersion contributions are unlikely to alter the qualitative conclusions about which interactions dominate the packing energetics. We note that more rigorous energy decomposition approaches such as symmetry-adapted perturbation theory (SAPT) can, in principle, offer slightly more accurate values for energy decomposition results for specific intermolecular interactions.^80,84^ However, such methods are not readily applicable to the present systems due to their open-shell electronic structure and size, which precludes their routine use in this context.

The dimerization energy of dimer B results from the stabilizing dispersive contribution, typical of hydrocarbon interactions. Dimer C has contributions from both dispersion and electronic energies, which is in agreement with the hydrogen bond and surrounding dispersion interactions. Dimers E and F both have repulsive electronic contributions that arise from the F···H and F···F electrostatic interactions; however, both are found to have overall negative dimerization energy, which arises from stabilizing dispersion forces. The binding energies of pairs A and D (which both involve stacking of the pseudosix-membered rings) are the greatest in magnitude and thus provide stability that is likely to be a key driving force for crystal packing. However, the methodology employed thus far does not supply detail about specific interactions, but rather all the interactions between the dimer pairs. For this reason, we decided to additionally study the role of specific dispersive and electronic interactions in dimer pairs A and D.

To investigate the electronic contribution of the dimerization energies of both systems, Mulliken charges were calculated in Crystal23 and are listed in the SI, while Figure 5 illustrates the partial charges of both Cu­(acac)_2_ (top), and Cu­(hfac)_2_ (bottom). In dimer pair A, the electronic contribution to the overall dimerization energy (+12.66 kJ mol^–1^) is not surprising because it is typical of eclipsed ring systems (sandwich conformation) that are usually electronically repulsive, as partially charged atoms are Coulombically repulsed by the atoms in the adjacent ring system.^85^ Small aromatic compounds like benzene (a prototypical system used to understand this phenomenon) will instead crystallize in a parallel displaced, or T-shaped structure, to maximize beneficial electrostatic interactions between the partially negative carbons and partially positive hydrogens.^86^ However, the acetylacetonate group exhibits alternating charges for each adjacent atom in the ring, as shown in Figure 5. The partial charges in Cu­(acac)_2_ are larger in magnitude than those of Cu­(hfac)_2_ due to the inductive effects of fluorine withdrawing electron density from the ring, which causes the atoms in the internal ring to become more neutral. These partial charges give rise to numerous Coulombic interactions in the solid state; in Figure 5, the Coulombic IMFs are indicated with black dotted lines. In Cu­(acac)_2_, the opposing partial charges within the eclipsing ring systems orient over each other. In contrast, the molecules in the Cu­(hfac)_2_ do not align their ring systems in a similar manner as Cu­(acac)_2_, indicating that these electronic interactions plays a role on the observed structures, and downstream properties of these crystals.

**Figure 5:**
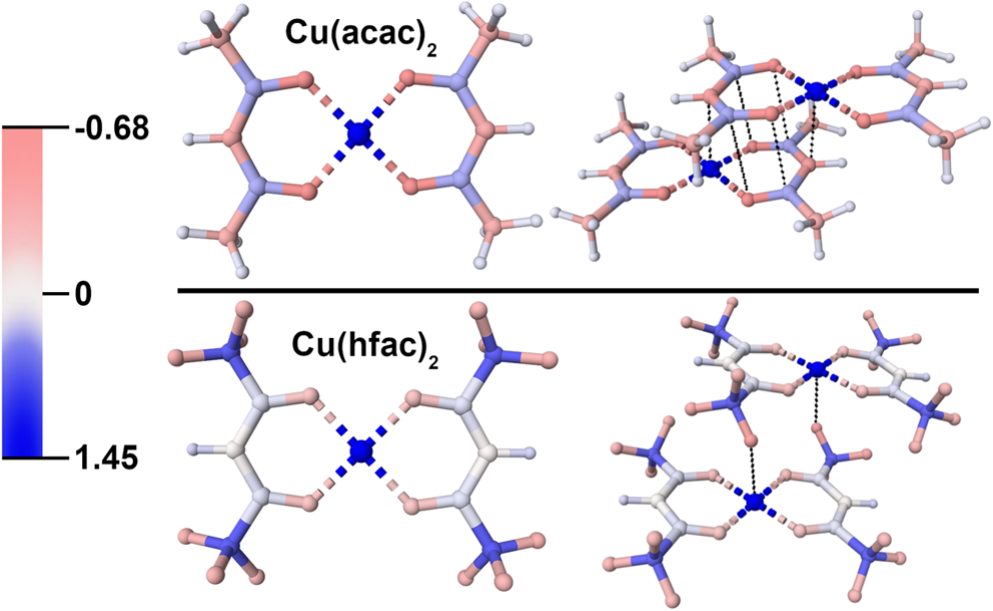
Charge maps of Cu­(acac)_2_ (top) and Cu­(hfac)_2_ (bottom) and electrostatic interactions of dimer pair A (top right) and D (bottom right). The color gradient is representative of the Mulliken charge values.

However, the electronic contribution (+12.66 kJ mol^–1^) of the total dimerization energy of Cu­(acac)_2_ dimer pair A (−47.54 kJ mol^–1^) is still unfavorable, and in order to understand the influence of the inductive effects of fluorine on the energy of this interaction, additional analyses were performed. In all dimer pairs investigated, the majority of the dimerization energy originated from dispersive interactions between the molecules (see Table 1). Methyl to trifluoromethyl substitution has a significant effect on the dispersive and electronic interactions because fluorine can withdraw electron density from the system, which both the electronic and dispersive interactions are dependent on. To investigate the impact of the electronegativity difference between hydrogen and fluorine on the downstream IMFs, hypothetical crystals were created computationally. The methyl groups in the Cu­(acac)_2_ crystal were replaced with trifluoromethyl groups (denoted TFM), and the trifluoromethyl groups of Cu­(hfac)_2_ were replaced with methyl groups (denoted M) while retaining the crystal structure of the original material, followed by a calculation of the electronic structure for the two systems. A second set of hypothetical systems were created, but this time hypothetical structures were subjected to a complete, unconstrained, geometry optimization using the same parameters as previous calculations. This was done to account for the different sizes of the elements (van der Waal radii of 1.20 and 1.47 Å for H and F, respectively). Dimer pairs A and D were extracted from these hypothetical crystals, and their dimerization energies were calculated. These values are presented in Table 2 and illustrated in Figure 6.

**Figure 6:**
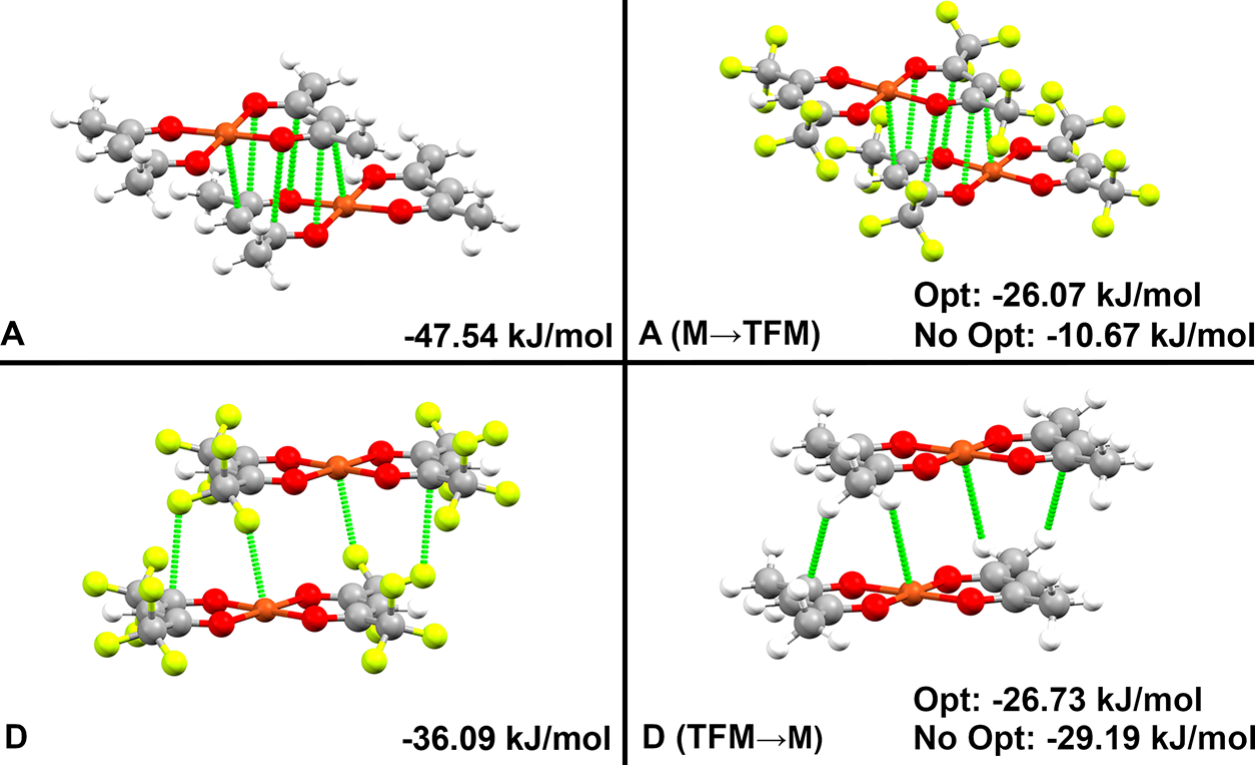
Dimer pairs extracted from the crystal structure are shown on the left, with the corresponding structures featuring computational functional group exchanges on the right. The top and bottom panels represent dimer pairs A and D, respectively. M and TFM denote methyl and trifluoromethyl, respectively.

**Table 2:** Dimerization Energies of Dimer Pairs Extracted from the Crystal Structures of Cu­(acac)_2_ and Cu­(hfac)_2_ ^a^

dimer pair	system	geometry	dimerization energy	electronic contribution	dispersion contribution
A	Cu(acac)_2_	optimized	–47.54	12.66	–60.19
	Cu(acac)_2_ w/TFM	unoptimized	–10.67	56.15	–66.82
		optimized	–26.07	32.05	–58.12
D	Cu(hfac)_2_	optimized	–36.09	1.74	–37.83
	Cu(hfac)_2_ w/M	unoptimized	–29.19	5.55	–34.74
		optimized	–26.73	19.48	–46.21

a Cu­(acac)_2_ w/trifluoromethyl and Cu­(hfac)_2_ w/methyl refer to the hypothetical crystals, that were optimized or extracted directly. All energies are reported in kJ mol^–1^.

The dispersive contribution of dimer pair A of Cu­(acac)_2_ was found to be −60.19 kJ mol^–1^, but after the computational substitution of M to TFM, this dispersive contribution became more stabilizing to −66.82 kJ mol^–1^. After allowing the unit cell to relax, this dispersive contribution becomes less stabilizing to −58.12 kJ mol^–1^. This demonstrates that the inductive effects of fluorine had little impact on the magnitude of dispersive forces between the molecules of dimer pair A. Likely the increase in electron density of the TFM group increased dispersive interactions between TFM groups of the adjacent molecules, but decreased the electron density, and thus the dispersive forces, between the two ring systems, effectively negating any significant beneficial stabilizing forces. However, there was a dramatic influence on the Coulombic interactions–as replacing the M to TFM increased the magnitude of destabilizing forces from +12.66 to +56.15 and +32.05 kJ mol^–1^ for the unoptimized and optimized structures, respectively. Despite the overall electronic contribution to dimerization energy being repulsive, the increase in energy upon substitution of M for TFM indicates that the overall electronic structure of the molecule largely influences the packing structure. Thus, it is clear that minimization of destabilizing electronic interactions results in the outsized role of dispersion forces in these crystals. Here, the dispersive forces that dominate crystal packing in Cu­(acac)_2_ likely also provide the crystal with the energetic flexibility to allow for the dissipation of external stress (bending) into these interactions–a possible origin of the unique dynamic effects reported for Cu­(acac)_2_.^48^

The dispersive contribution of the dimerization energy of Cu­(hfac)_2_ for dimer pair D was found to be −37.83 kJ mol^–1^, while the TFM to M substitution produced a dispersive contribution of −34.74 and −46.21 kJ mol^–1^ after optimization. Here, the magnitude of the dispersive contributions are, once again, not largely affected by the TFM to M substitution. However, the electronic contributions of both systems are largely influenced by the computational substitution. The destabilizing electronic contribution (+1.74 kJ mol^–1^), increased upon substitution to +5.55 and +19.48 kJ mol^–1^ for the unoptimized and optimized structures, respectively. In Cu­(hfac)_2_, the partially negative fluorine atoms orient above and below the copper cation, producing a stabilizing Coulombic interaction. The F···Cu···F interaction in Cu­(hfac)_2_ is similar to the alignment of partial charges in Cu­(acac)_2_ dimer A because they are both favorable electronic interactions within an overall unfavorable electronic environment. These favorable interactions sufficiently lower the destabilizing electronic energy compared to the TFM and M hypothetical systems, allowing dispersive forces to dominate and dictate crystal packing.

## 4 Conclusions

Overall, the pairing of terahertz vibrational spectroscopy, static X-ray diffraction, and ss-DFT provided complementary insight into the bulk manifestation (packing structure) of the intermolecular forces in two molecular crystals, Cu­(acac)_2_ and Cu­(hfac)_2_. THz-TDS enabled direct sampling of intermolecular vibrational dynamics, which provides quantitative information related to the strength of various IMFs. The interplay of IMFs and low-frequency dynamics was visualized through the animation of the observed vibrational normal modes. Using ss-DFT simulations, quantitative insight into each specific interaction and their associated energetic origins enables pinpointing the IMFs that promote (and hinder) the formation of specific crystal forms. This energetic analysis highlighted the delicate interplay between pure Coulombic interactions and dispersive forces, with the domination of van der Waals interactions providing the strongest evidence for the various properties investigated. This work emphasizes the interplay between intermolecular interactions, three-dimensional structure, and lattice dynamics, validating the combination of THz-TDS and ss-DFT as a powerful strategy for an in-depth understanding of IMFs.

## Supplementary Material







## Notes

Electrostatic intermolecular interactions; Mulliken charge analyses; terahertz time-domain spectroscopy (THz-TDS) spectra with comparison to DFT-predicted phonon modes; detailed vibrational mode assignments and symmetry labels; powder X-ray diffraction (PXRD) data for anhydrous materials and hydrates; and experimental THz-TDS spectra tracking the hydration state of Cu­(hfac)_2_ samples (PDF)
Vibration animations (ZIP)
Calculations (ZIP)
