# Supplementary material for: Interrogation of the Intermolecular Forces That Drive Bulk Properties of Molecular Crystals with Terahertz Spectroscopy and Density Functional Theory
Source: Cryst Growth Des. 2025 May 23;25(11):3697–706. doi: 10.1021/acs.cgd.5c00007 (PMC12148306; doi:10.1021/acs.cgd.5c00007)
Supplement: Supplementary file 1 [file cg5c00007_si_001.pdf]

Interrogation of the Intermolecular Forces that Drive Bulk Properties in Molecular Crystals with Terahertz Spectroscopy and Density Functional Theory

William B. Stoll,[†] Peter A. Banks,[†,‡] Steven G. Dannenberg,[¶] Rory Waterman,[¶] Luca Catalano,[†,§] and Michael T. Ruggiero[*,,†]

† *Department of Chemistry, University of Rochester, Rochester, NY 14627, USA*

‡ *Department of Chemistry, University of Mons, Mons, Belgium*

¶ *Department of Chemistry, University of Vermont, Burlington, VT 05405, USA*

§ *Department of Life Sciences, University of Modena and Reggio Emilia, Modena 41125, Italy*

E-mail: Michael.Ruggiero@rochester.edu

Contents

1 $Cu(hfac)_2$ Cu-F interaction **2**

2 Mulliken Charges **3**

3 Phonon Modes **4**

4 Powder X-ray Diffraction **17**

5 THz-TDS of $Cu(hfac)_2$ Hydrates **20**

1 Cu(hfac)$_2$ Cu-F interaction

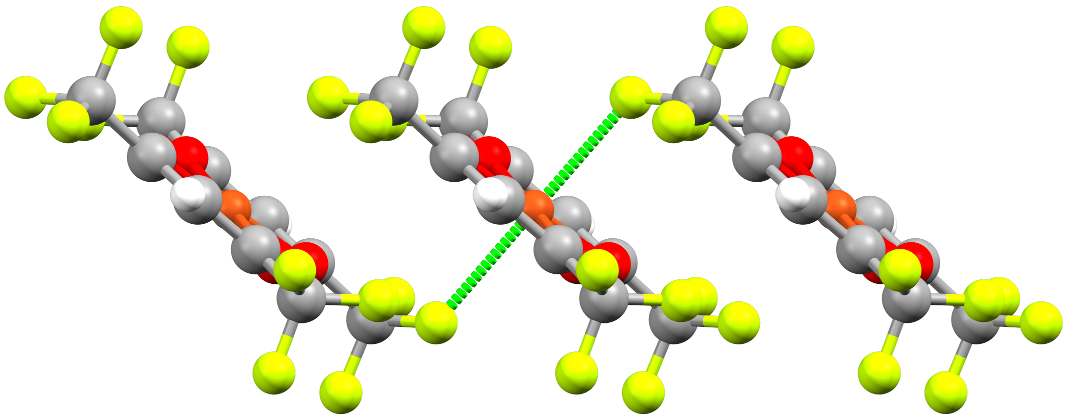

Figure S1: The F$\cdots$Cu$\cdots$F electrostatic interaction observed in the Cu(hfac)$_2$ crystal structure.

2 Mulliken Charges

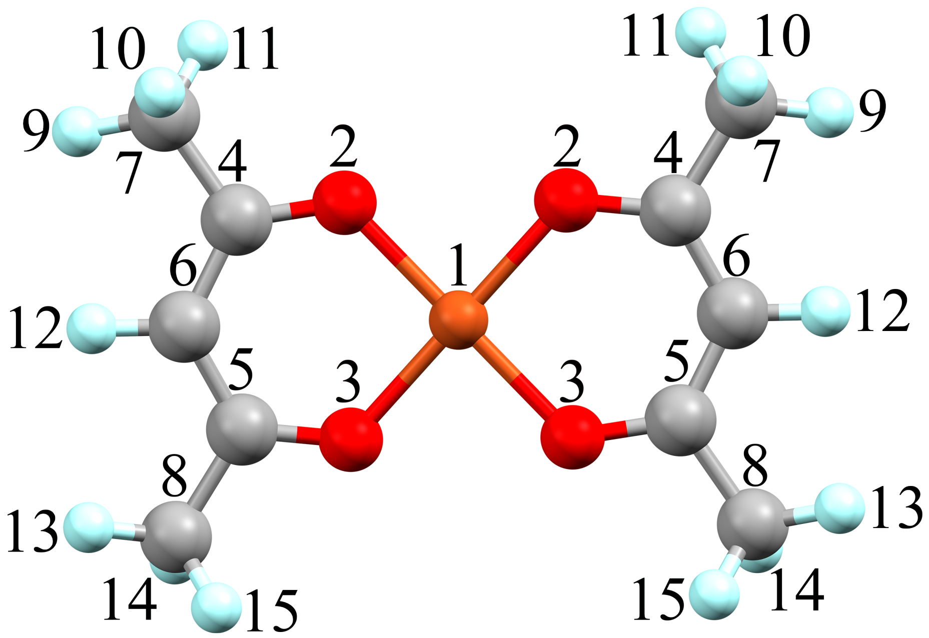

Figure S2: Atom labels for **Table S1**, the blue atoms can either be H or F for $Cu(acac)_2$ and $Cu(hfac)_2$ respectively. Atom 12 is always a hydrogen.

Table S1: Mulliken charges for $Cu(acac)_2$ and $Cu(hfac)_2$, where the atom label corresponds to the atom number in **Figure S2**.

**Atom Label**	**1**	**2**	**3**	**4**	**5**	**6**	**7**	**8**	**9**	**10**	**11**	**12**	**13**	**14**	**15**
$Cu(acac)_2$	1.420	-0.683	-0.686	0.361	0.361	-0.167	-0.138	-0.137	0.071	0.063	0.046	0.022	0.069	0.046	0.062
$Cu(hfac)_2$	1.451	-0.614	-0.625	0.271	0.270	-0.133	0.890	0.887	-0.304	-0.294	-0.292	0.109	-0.291	-0.299	-0.301

3 Phonon Modes

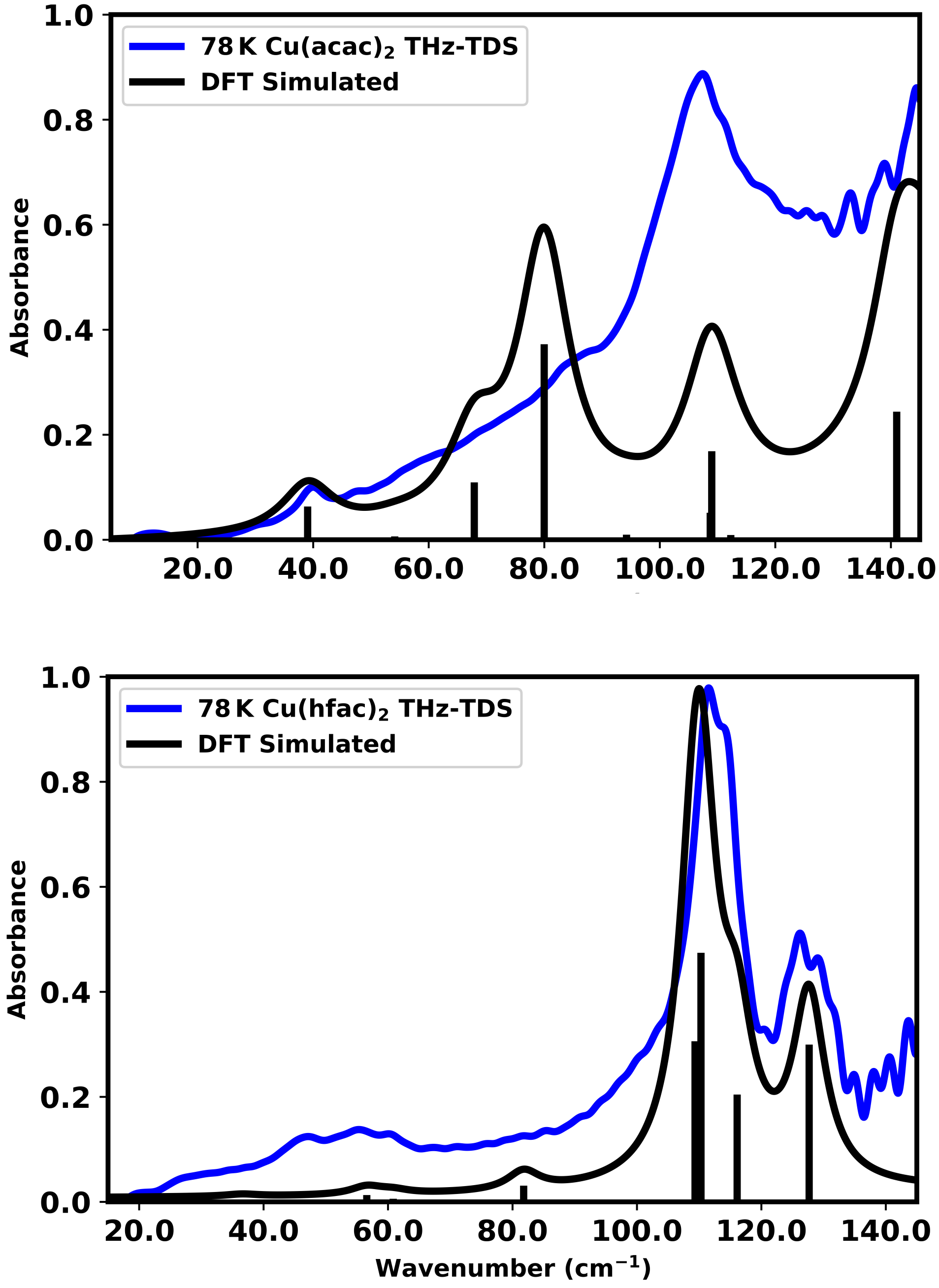

Figure S3: Experimental 78 K THz-TDS spectra (blue) and ss-DFT predicted spectra (black) of Cu(acac)$_2$ (Top) and Cu(hfac)$_2$ (Bottom). The simulated spectra shown here have had their frequencies scaled by 0.9 to account for the difference in temperature between the experiment and the effective 0 K temperature of the DFT simulations.

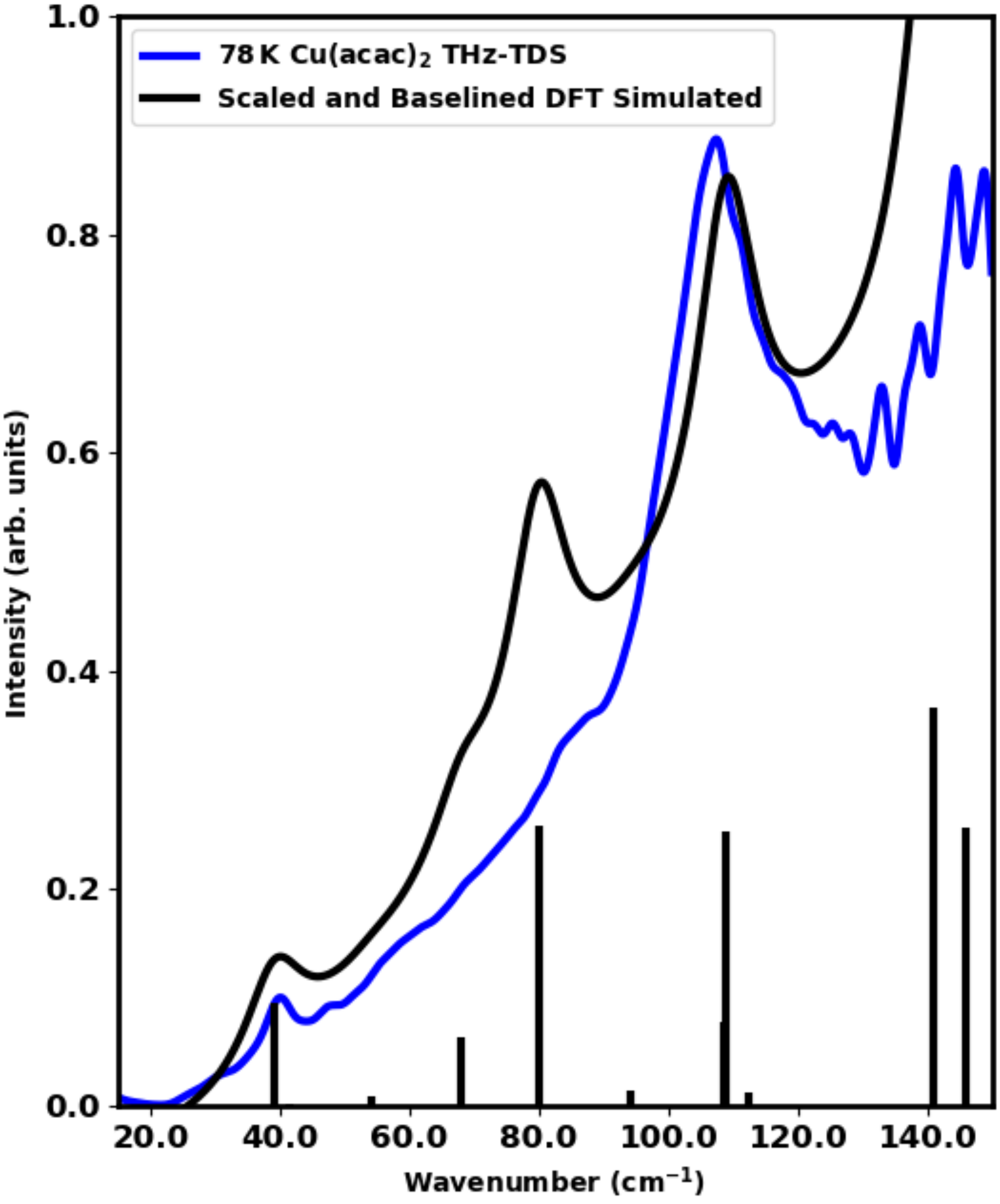

Figure S4: Experimental 78 K THz-TDS spectra (blue) of $Cu(acac)_2$ (top). The background absorption in the experimental spectrum was added to the scaled DFT-simulated spectrum to produce the black curve. This background is likely caused by scattering (from either the crystalline particles or porous air pockets in the pellet).

Table S2: Phonon mode descriptions for $Cu(acac)_2$. Calculated frequencies are in wavenumbers.

**Frequency (cm^{-1})**	**Mode Description**
43.39	Sliding of sheets along b, methyl torsion
75.43	Sliding of sheets along c, methyl torsion
88.87	In-plane rocking, methyl torsion
120.81	Entire molecule in-plane twisting, wagging of H
121.09	Entire molecule scissoring

Table S3: Phonon mode descriptions for $Cu(hfac)_2$. Calculated frequencies are in wavenumbers.

**Frequency (cm^{-1})**	**Mode Description**
40.59	Acetylacetonate deformation around c, displacement of oxygen and copper out of (011) plane, TFM torsion
62.86	TFM torsion, displacement of oxygen and copper out of (011) plane
67.59	Scissoring of entire molecule
90.89	Twisting of entire molecule perpendicular to (011) plane, TFM torsion
121.48	Twisting of entire molecule diagonal to the (001) plane, TFM torsion
122.54	Twisting of entire molecule perpendicular to (011) plane
129.01	Displacement of oxygen and copper out of (011) plane, rocking of fluorines
141.81	Twist of oxygens, acetylacetonate deformation

Table S4: DFT calculated vibrational modes for $Cu(acac)_2$, I and A stand for inactive and active respectively.

**Mode**	**Freq. (cm^{-1})**	**Symmetry**	**IR Activity**	**Intensity (km mol^{-1})**	**Raman Activity**
1	0	Au	A	0	I
2	0	Bu	A	0	I
3	0	Bu	A	0	I
4	50.8793	Au	A	0.02	I
5	51.4378	Bu	A	1.16	I
6	59.1088	Bg	I	0	A
7	67.7816	Au	A	0.04	I
8	71.2445	Ag	I	0	A

9	87.8238	Ag	I	0	A
10	96.0291	Au	A	1.54	I
11	102.0692	Bu	A	10.53	I
12	104.0071	Ag	I	0	A
13	105.4724	Bg	I	0	A
14	107.2671	Bg	I	0	A
15	110.3613	Au	A	1.48	I
16	124.1004	Bu	A	3.38	I
17	127.0028	Au	A	1.76	I
18	129.0587	Bu	A	0.53	I
19	132.2832	Ag	I	0	A
20	144.1812	Bg	I	0	A
21	160.8799	Au	A	15.62	I
22	163.6234	Ag	I	0	A
23	165.1701	Bg	I	0	A
24	173.8503	Bu	A	4.71	I
25	176.8702	Ag	I	0	A
26	182.5874	Bg	I	0	A
27	198.2046	Bu	A	50.55	I
28	200.3335	Bg	I	0	A
29	201.5441	Ag	I	0	A
30	201.6053	Au	A	6.32	I
31	202.5378	Bu	A	9.29	I
32	207.2513	Au	A	0.09	I
33	215.8177	Au	A	39.24	I
34	217.2772	Bg	I	0	A

35	220.1194	Bu	A	0.8	I
36	224.738	Ag	I	0	A
37	228.9256	Bg	I	0	A
38	229.0902	Ag	I	0	A
39	240.6389	Ag	I	0	A
40	243.934	Bu	A	71.75	I
41	247.5956	Au	A	194.74	I
42	259.3498	Bg	I	0	A
43	293.8526	Au	A	34.69	I
44	294.0862	Bu	A	9.32	I
45	301.4882	Bg	I	0	A
46	301.7151	Ag	I	0	A
47	317.2245	Bu	A	50.13	I
48	318.7785	Au	A	29.24	I
49	356.0594	Au	A	0.46	I
50	365.0587	Bu	A	1.38	I
51	420.6922	Ag	I	0	A
52	427.3643	Bg	I	0	A
53	449.4444	Bu	A	51.83	I
54	451.6732	Au	A	0.14	I
55	463.7431	Bg	I	0	A
56	471.1025	Ag	I	0	A
57	481.7442	Bu	A	94.29	I
58	482.5506	Au	A	77.57	I
59	568.187	Bg	I	0	A
60	569.9414	Ag	I	0	A

61	571.9085	Au	A	2.15	I
62	580.3198	Bu	A	8.85	I
63	591.1576	Ag	I	0	A
64	594.342	Bg	I	0	A
65	633.2508	Bu	A	197.48	I
66	634.3887	Au	A	0.01	I
67	669.9393	Bu	A	89.62	I
68	675.4549	Ag	I	0	A
69	675.6067	Au	A	81.36	I
70	678.5633	Bg	I	0	A
71	707.2917	Au	A	11.7	I
72	709.8704	Bu	A	45.89	I
73	712.5909	Ag	I	0	A
74	713.2611	Bg	I	0	A
75	831.5173	Ag	I	0	A
76	833.2213	Au	A	165.93	I
77	843.2795	Bg	I	0	A
78	843.3294	Bu	A	113.12	I
79	976.5268	Au	A	39.82	I
80	978.5052	Ag	I	0	A
81	979.4354	Bu	A	26.85	I
82	980.3747	Ag	I	0	A
83	980.4484	Bg	I	0	A
84	983.8609	Bg	I	0	A
85	986.396	Bu	A	92.12	I
86	989.4107	Au	A	4.67	I

87	1035.9407	Bu	A	105.74	I
88	1037.1213	Ag	I	0	A
89	1038.0857	Au	A	103.13	I
90	1040.3837	Bg	I	0	A
91	1059.8744	Bg	I	0	A
92	1061.7414	Au	A	26.1	I
93	1062.9628	Bu	A	45.77	I
94	1063.224	Ag	I	0	A
95	1066.0978	Bu	A	27.92	I
96	1067.0472	Ag	I	0	A
97	1067.3735	Bg	I	0	A
98	1068.6672	Au	A	0.85	I
99	1072.5643	Au	A	58.75	I
100	1075.4647	Bu	A	74.75	I
101	1078.4298	Ag	I	0	A
102	1078.6524	Bg	I	0	A
103	1226.4564	Bg	I	0	A
104	1226.556	Ag	I	0	A
105	1228.0783	Bu	A	69.57	I
106	1231.0034	Au	A	2.36	I
107	1329.3546	Au	A	45.88	I
108	1335.921	Ag	I	0	A
109	1336.1764	Bu	A	84.06	I
110	1340.987	Bg	I	0	A
111	1395.0335	Ag	I	0	A
112	1396.5139	Bu	A	883.08	I

113	1397.2086	Au	A	0.66	I
114	1402.1479	Au	A	21.77	I
115	1404.9381	Bg	I	0	A
116	1412.6377	Bu	A	187.41	I
117	1412.8697	Ag	I	0	A
118	1414.7485	Bg	I	0	A
119	1431.0252	Ag	I	0	A
120	1440.3903	Bg	I	0	A
121	1460.2077	Au	A	32.86	I
122	1462.76	Bu	A	903.83	I
123	1469.7424	Ag	I	0	A
124	1469.8211	Bg	I	0	A
125	1470.9708	Au	A	9.31	I
126	1472.5286	Bu	A	261.65	I
127	1478.9376	Ag	I	0	A
128	1486.4959	Au	A	338.29	I
129	1501.559	Bu	A	88.06	I
130	1503.1178	Bg	I	0	A
131	1504.701	Ag	I	0	A
132	1510.5727	Bg	I	0	A
133	1511.9149	Au	A	0.06	I
134	1513.3722	Ag	I	0	A
135	1524.4781	Bu	A	5.78	I
136	1525.6356	Bg	I	0	A
137	1530.0999	Au	A	42.56	I
138	1542.8387	Bu	A	47.9	I

139	1579.0988	Ag	I	0	A
140	1593.7209	Bu	A	2506.84	I
141	1594.059	Au	A	28.6	I
142	1596.4688	Bg	I	0	A
143	1633.7959	Bu	A	1221.8	I
144	1637.9046	Au	A	2232.24	I
145	1638.7016	Ag	I	0	A
146	1648.4012	Bg	I	0	A
147	3078.8781	Ag	I	0	A
148	3079.1293	Au	A	0.19	I
149	3079.8534	Bg	I	0	A
150	3079.9355	Bu	A	22.49	I
151	3083.4638	Au	A	53.57	I
152	3083.5644	Bu	A	3.73	I
153	3083.8422	Ag	I	0	A
154	3083.893	Bg	I	0	A
155	3165.169	Bu	A	82.34	I
156	3165.4174	Au	A	0.32	I
157	3167.0083	Bg	I	0	A
158	3167.0184	Ag	I	0	A
159	3176.9807	Bu	A	1.76	I
160	3177.0197	Bg	I	0	A
161	3177.2688	Ag	I	0	A
162	3177.3414	Au	A	31.04	I
163	3188.5902	Au	A	3.61	I
164	3189.1825	Bu	A	22.73	I

165	3189.3942	Ag	I	0	A
166	3190.2793	Bg	I	0	A
167	3221.7467	Bu	A	39.13	I
168	3221.8951	Bg	I	0	A
169	3224.0465	Au	A	21.82	I
170	3224.0985	Ag	I	0	A
171	3248.1645	Bu	A	2.88	I
172	3248.2082	Bg	I	0	A
173	3248.8023	Au	A	9.08	I
174	3248.9093	Ag	I	0	A

Table S5: DFT calculated vibrational modes for $Cu(hfac)_2$, I and A stand for inactive and active respectively

**Mode**	**Freq. (cm^{-1})**	**Symmetry**	**IR Activity**	**Intensity (km mol^{-1})**	**Raman Activity**
1	0	Au	A	0	I
2	0	Au	A	0	I
3	0	Au	A	0	I
4	32.2486	Ag	I	0	A
5	41.6232	Au	A	0.09	I
6	48.9684	Ag	I	0	A
7	58.7449	Ag	I	0	A
8	63.9426	Au	A	0.26	I
9	69.2971	Au	A	0.09	I
10	78.8496	Ag	I	0	A
11	93.8794	Au	A	0.39	I
12	95.6362	Ag	I	0	A

13	111.5374	Ag	I	0	A
14	114.1636	Ag	I	0	A
15	125.9078	Au	A	6.39	I
16	126.9991	Au	A	6.55	I
17	130.1841	Au	A	6.57	I
18	130.6248	Ag	I	0	A
19	138.0386	Ag	I	0	A
20	146.1787	Au	A	6.89	I
21	157.7156	Ag	I	0	A
22	184.9217	Ag	I	0	A
23	223.1891	Au	A	2.29	I
24	231.5723	Au	A	51.59	I
25	257.5448	Ag	I	0	A
26	262.7417	Au	A	6.81	I
27	267.0704	Ag	I	0	A
28	279.8252	Au	A	4.14	I
29	282.1562	Au	A	3.63	I
30	282.3077	Ag	I	0	A
31	298.8208	Au	A	75.44	I
32	327.5066	Ag	I	0	A
33	335.9672	Au	A	16.51	I
34	365.6403	Ag	I	0	A
35	365.8524	Au	A	3.11	I
36	394.7525	Ag	I	0	A
37	425.8831	Au	A	2.17	I
38	489.1433	Ag	I	0	A

39	512.0661	Au	A	1.19	I
40	512.828	Ag	I	0	A
41	528.5647	Ag	I	0	A
42	528.773	Au	A	36.01	I
43	530.2293	Ag	I	0	A
44	532.0942	Au	A	13.78	I
45	536.7102	Au	A	15.5	I
46	592.4495	Ag	I	0	A
47	607.1975	Ag	I	0	A
48	608.6135	Au	A	159.82	I
49	615.9243	Au	A	31.91	I
50	680.9786	Ag	I	0	A
51	690.2865	Au	A	229.15	I
52	744.9962	Au	A	8.27	I
53	745.6934	Ag	I	0	A
54	755.1876	Au	A	19.96	I
55	756.3174	Ag	I	0	A
56	792.19	Ag	I	0	A
57	794.2942	Au	A	3.64	I
58	834.7198	Ag	I	0	A
59	837.1235	Au	A	129.14	I
60	840.9814	Ag	I	0	A
61	842.5454	Au	A	224.93	I
62	983.0393	Au	A	1.54	I
63	984.7876	Ag	I	0	A
64	1138.3198	Au	A	124.35	I

65	1144.3696	Ag	I	0	A
66	1177.8088	Au	A	658.42	I
67	1178.5427	Ag	I	0	A
68	1191.8234	Ag	I	0	A
69	1198.2569	Au	A	1491.81	I
70	1203.1551	Ag	I	0	A
71	1213.2482	Au	A	479.28	I
72	1218.7253	Au	A	1579.37	I
73	1231.9671	Ag	I	0	A
74	1248.6101	Au	A	1163.81	I
75	1255.5872	Ag	I	0	A
76	1276.6201	Au	A	1097.79	I
77	1288.0084	Ag	I	0	A
78	1397.1994	Au	A	35.76	I
79	1399.746	Ag	I	0	A
80	1505.5023	Ag	I	0	A
81	1518.7135	Au	A	982.94	I
82	1616.0541	Au	A	620.07	I
83	1617.2757	Ag	I	0	A
84	1699.4783	Au	A	961.13	I
85	1718.6789	Ag	I	0	A
86	3312.7452	Au	A	84.44	I
87	3313.189	Ag	I	0	A

4 Powder X-ray Diffraction

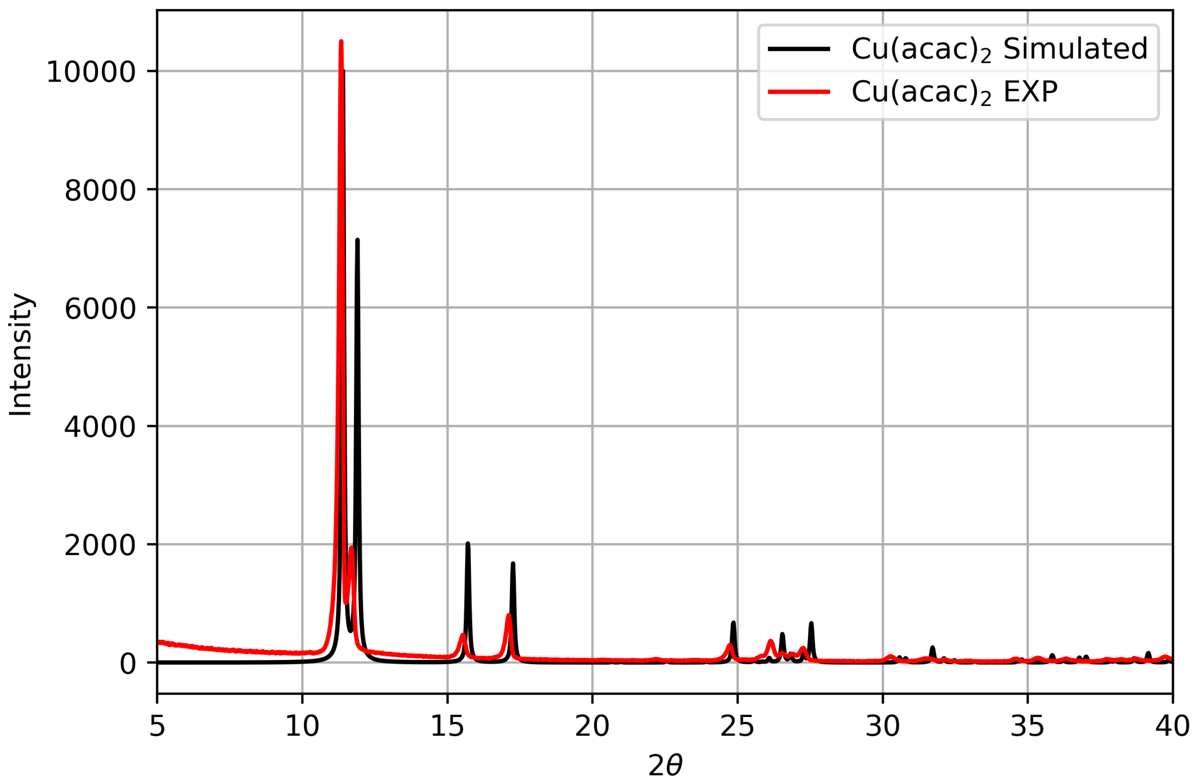

Figure S5: Power X-ray diffraction data for as-received Cu(acac)$_2$, compared to a simulated pattern generated from single-crystal diffraction data.

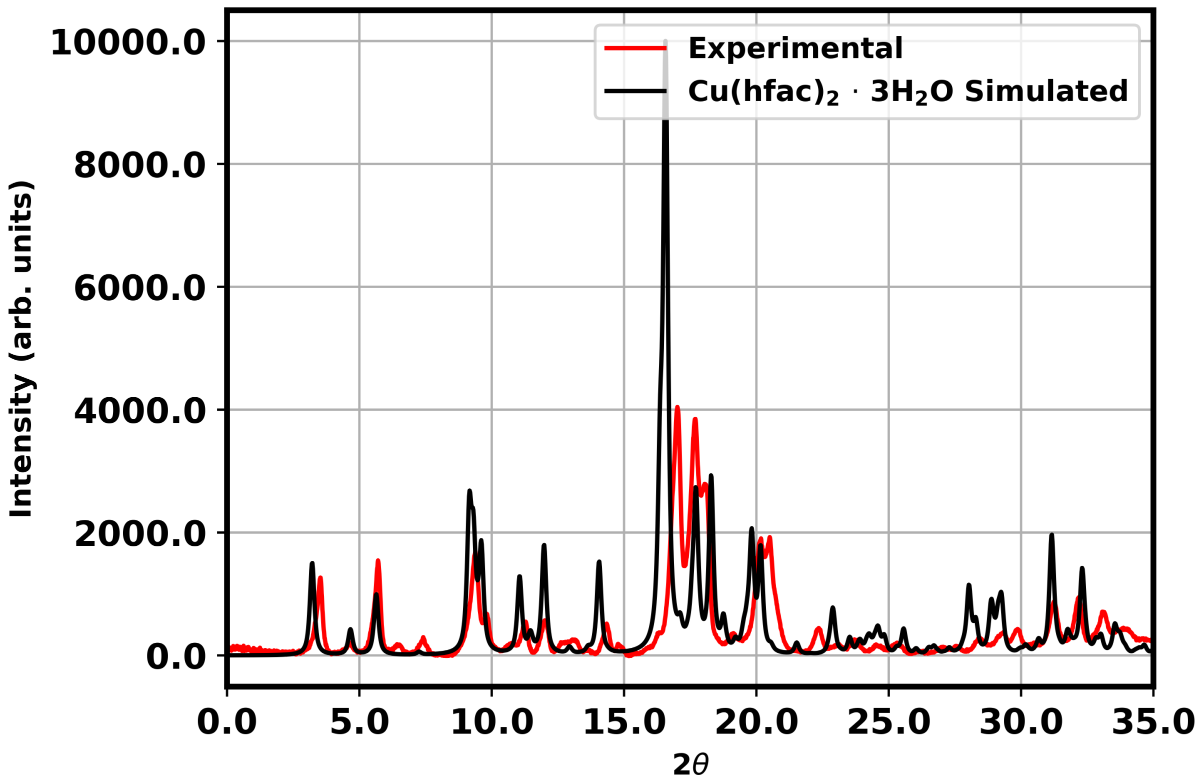

Figure S6: Power X-ray diffraction data for as-received $Cu(hfac)_2 \cdot 3H_2O$, compared to a simulated pattern generated from single-crystal diffraction data.

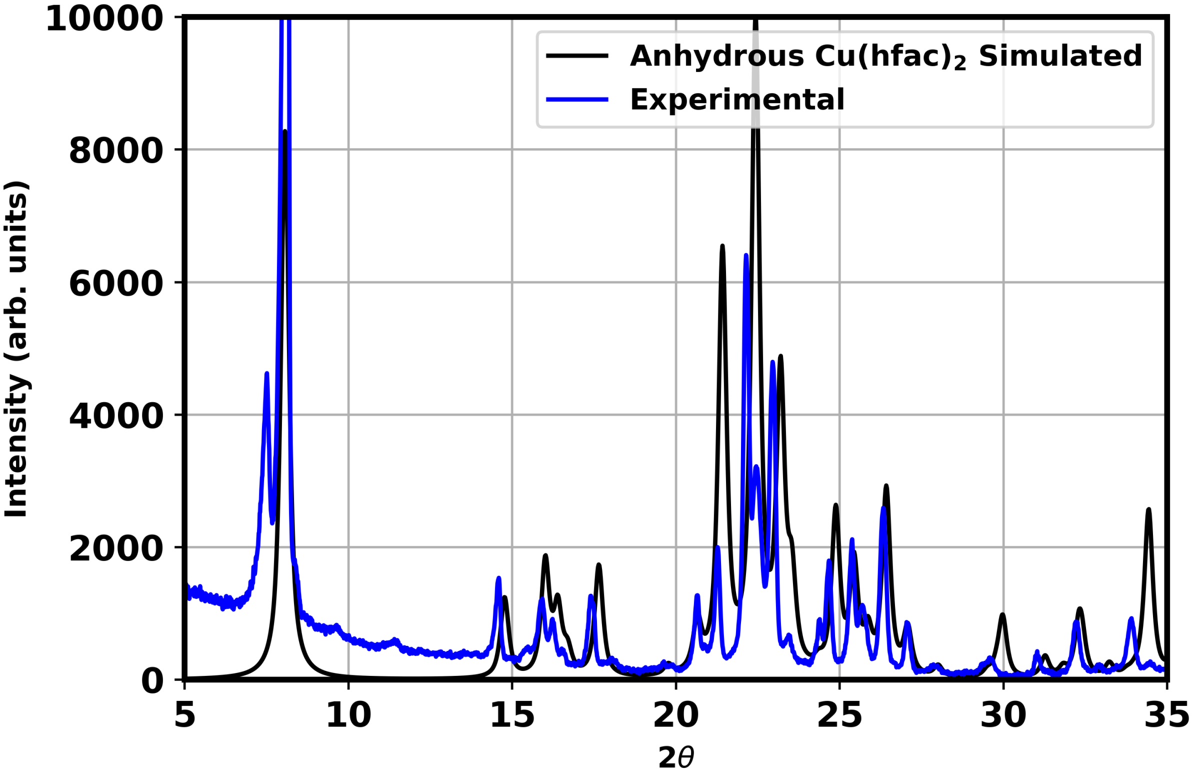

Figure S7: Power X-ray diffraction data for dried anhydrous Cu(hfac)$_2$, compared to a simulated pattern generated from single-crystal diffraction data.

5 THz-TDS of $Cu(hfac)_2$ Hydrates

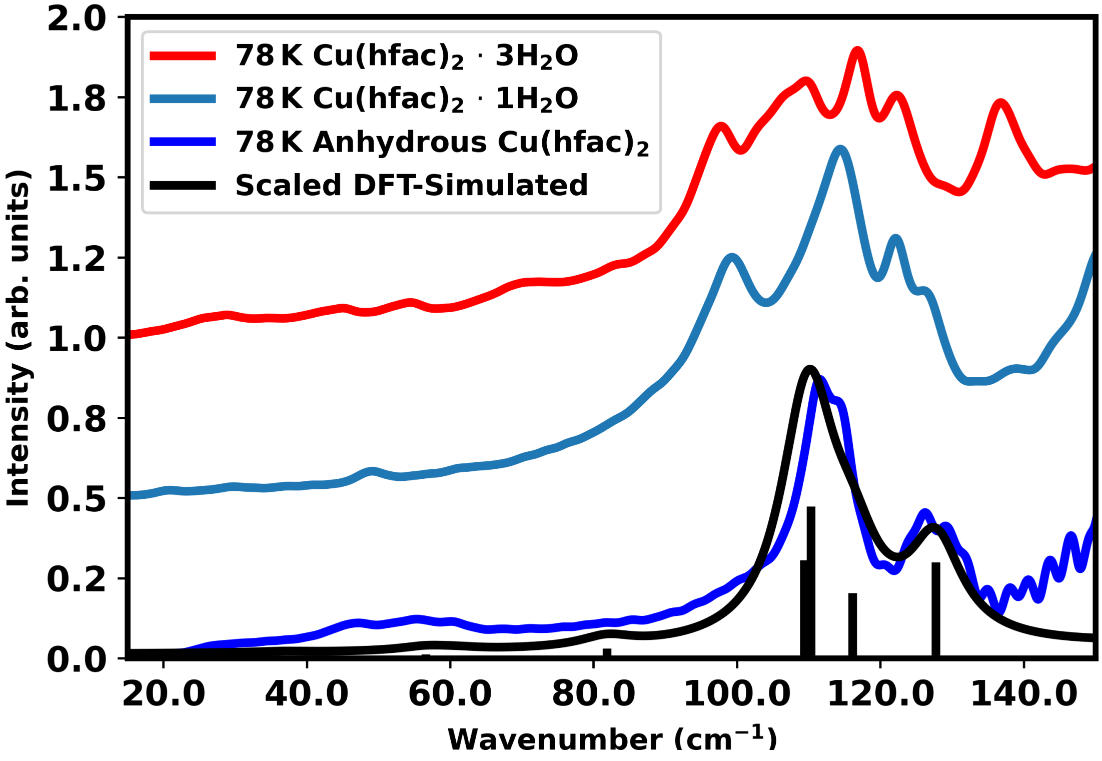

Figure S8: THz-TDS Experiments of $Cu(hfac)_2$ as a function of drying from the as-received trihydrate sample. The DFT-simulated spectrum has been scaled by 0.9
