# Supplementary figures and images for: Interrogation of the Intermolecular Forces That Drive Bulk Properties of Molecular Crystals with Terahertz Spectroscopy and Density Functional Theory
Source: Cryst Growth Des. 2025 May 23;25(11):3697–706. doi: 10.1021/acs.cgd.5c00007 (PMC12148306; doi:10.1021/acs.cgd.5c00007)

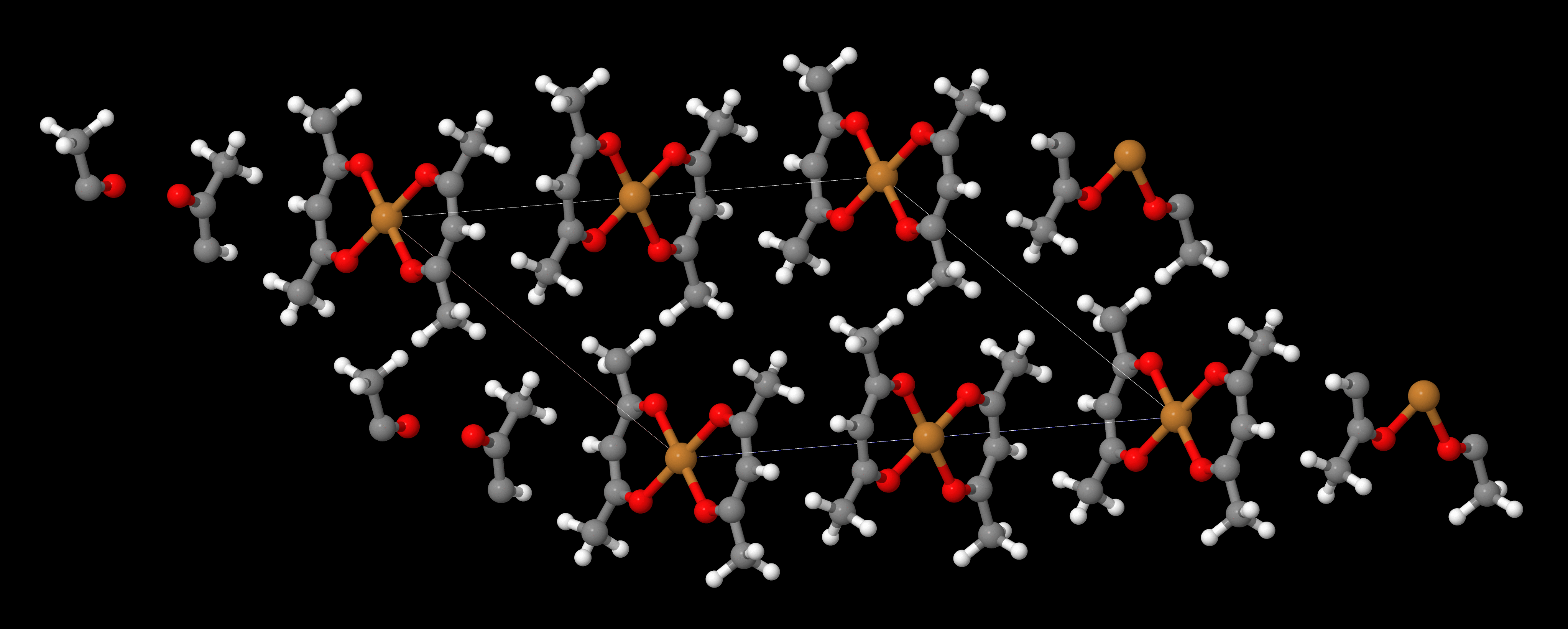

Supplement: Supplementary file 2 [file cg5c00007_si_002.zip › Vibration_Animations/CuAcAc/43.39.gif]

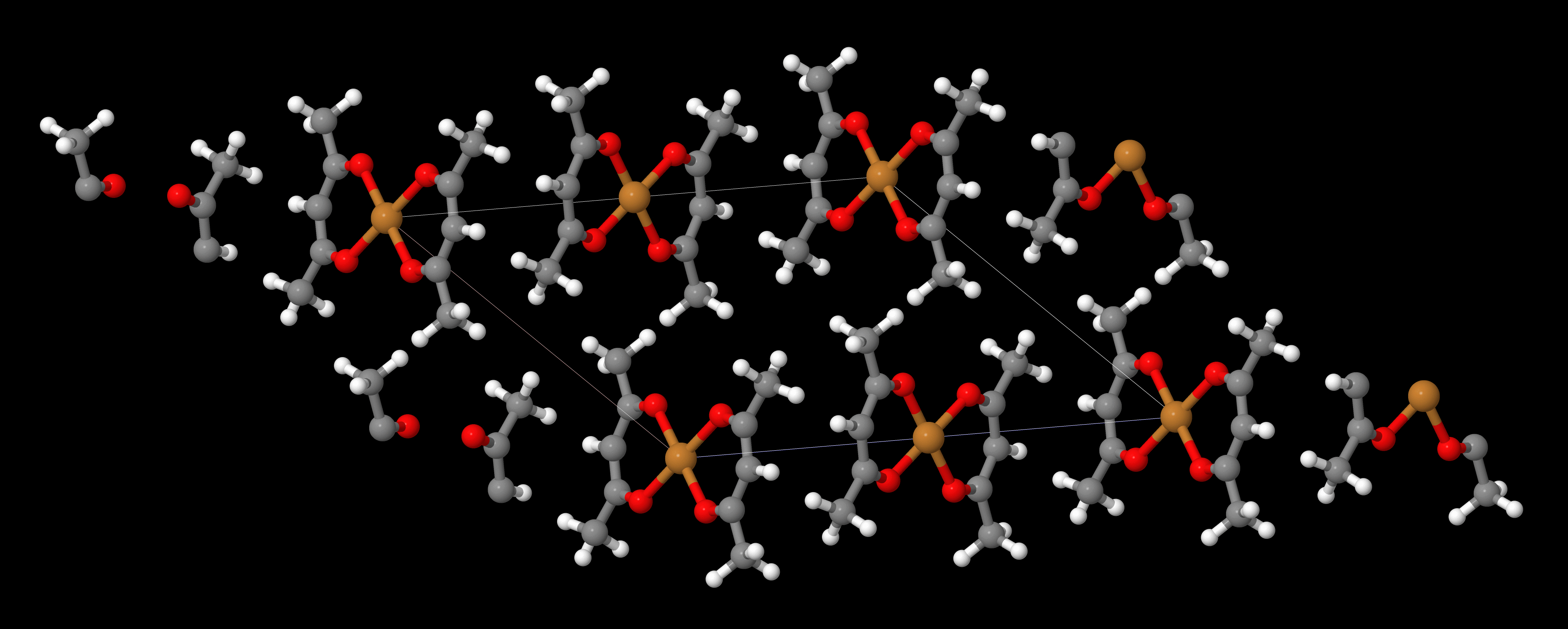

Supplement: Supplementary file 2 [file cg5c00007_si_002.zip › Vibration_Animations/CuAcAc/121.09.gif]

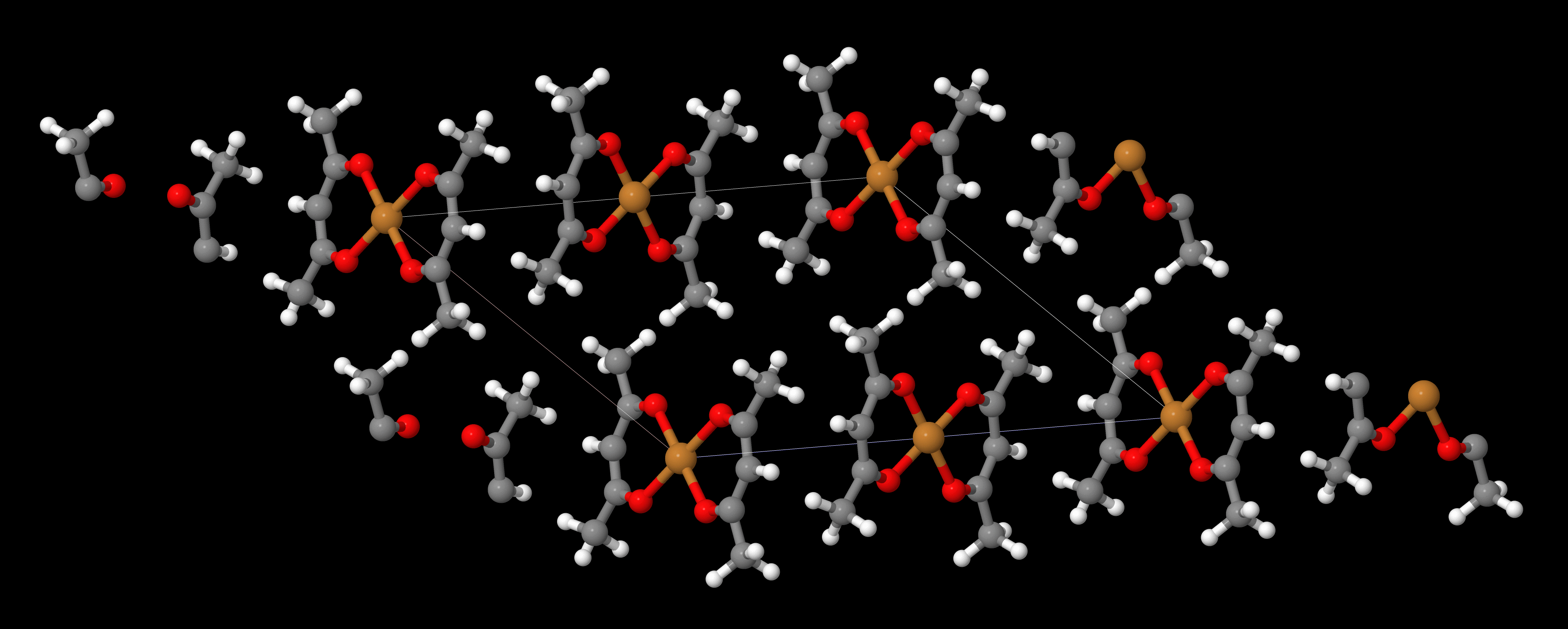

Supplement: Supplementary file 2 [file cg5c00007_si_002.zip › Vibration_Animations/CuAcAc/75.43.gif]

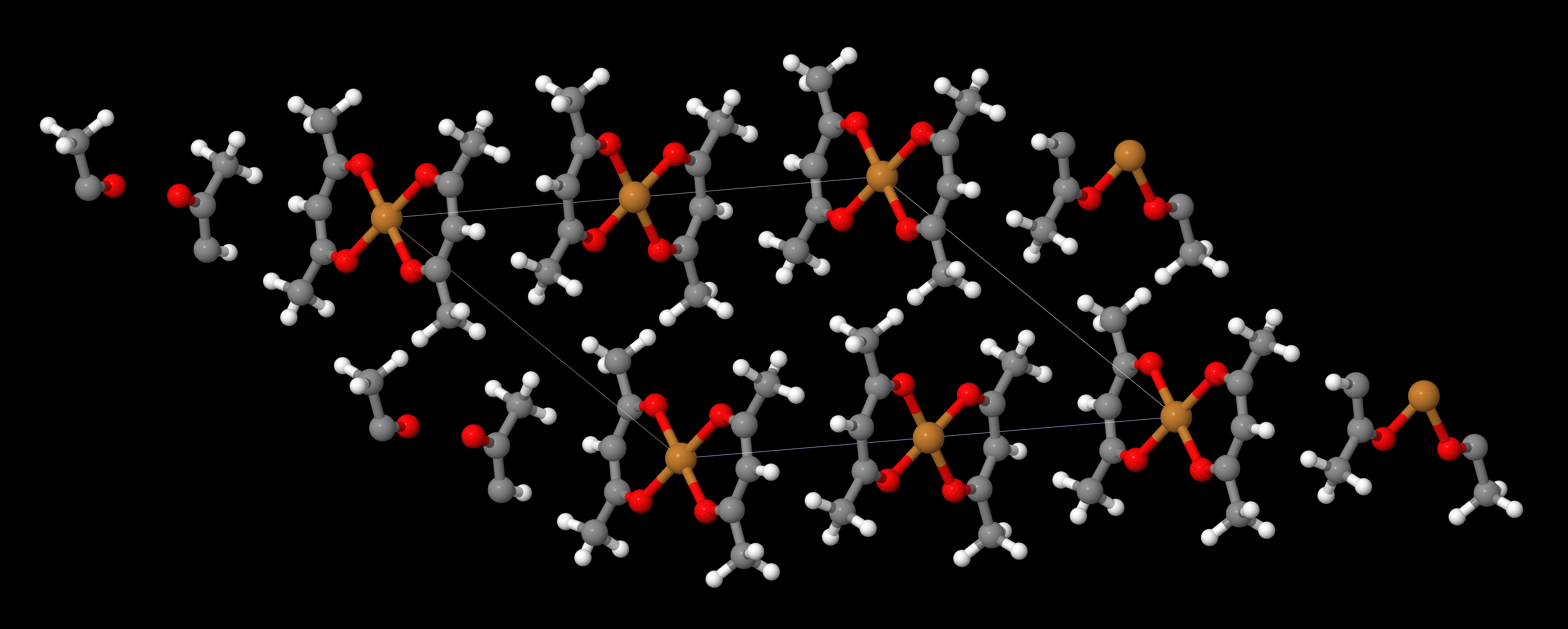

Supplement: Supplementary file 2 [file cg5c00007_si_002.zip › Vibration_Animations/CuAcAc/88.87.gif]

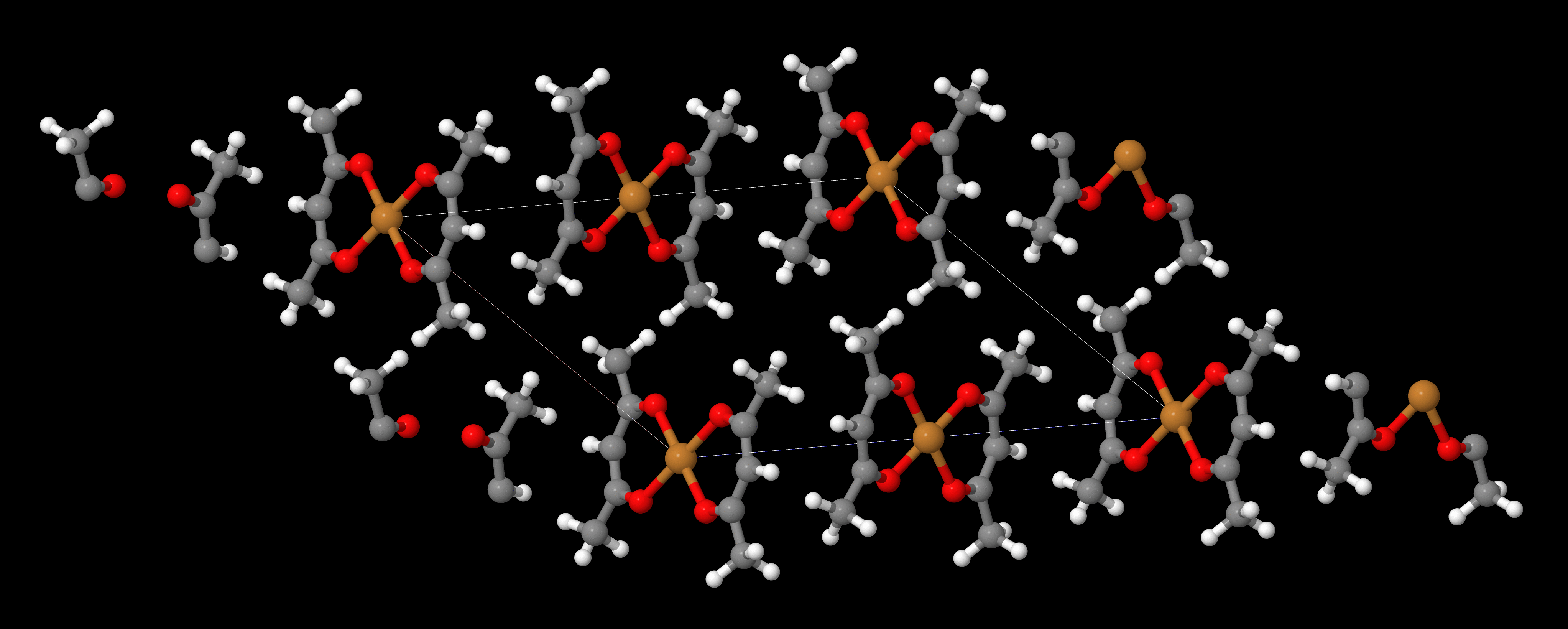

Supplement: Supplementary file 2 [file cg5c00007_si_002.zip › Vibration_Animations/CuAcAc/120.81.gif]

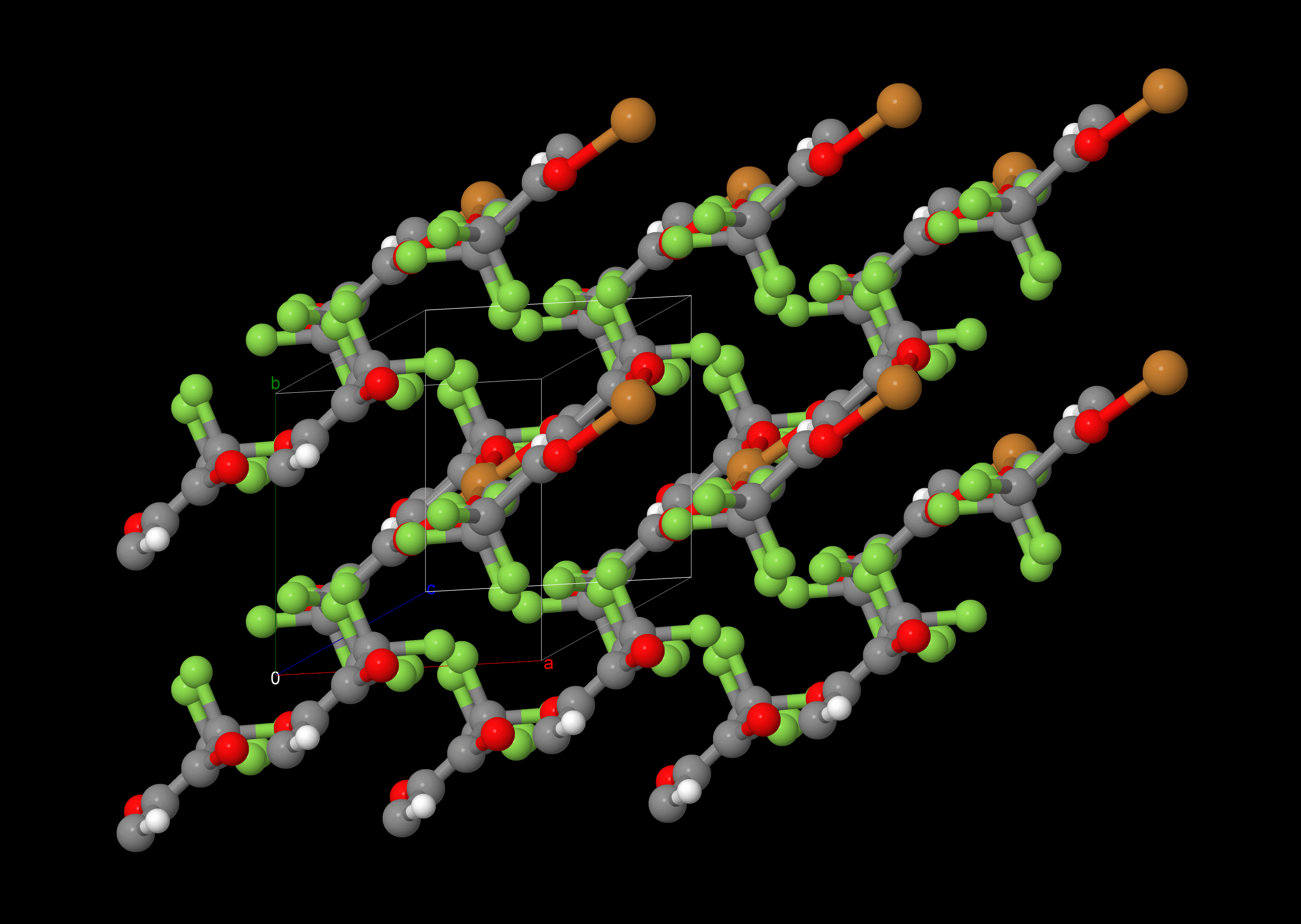

Supplement: Supplementary file 2 [file cg5c00007_si_002.zip › Vibration_Animations/CuAcAcF/90.89.gif]

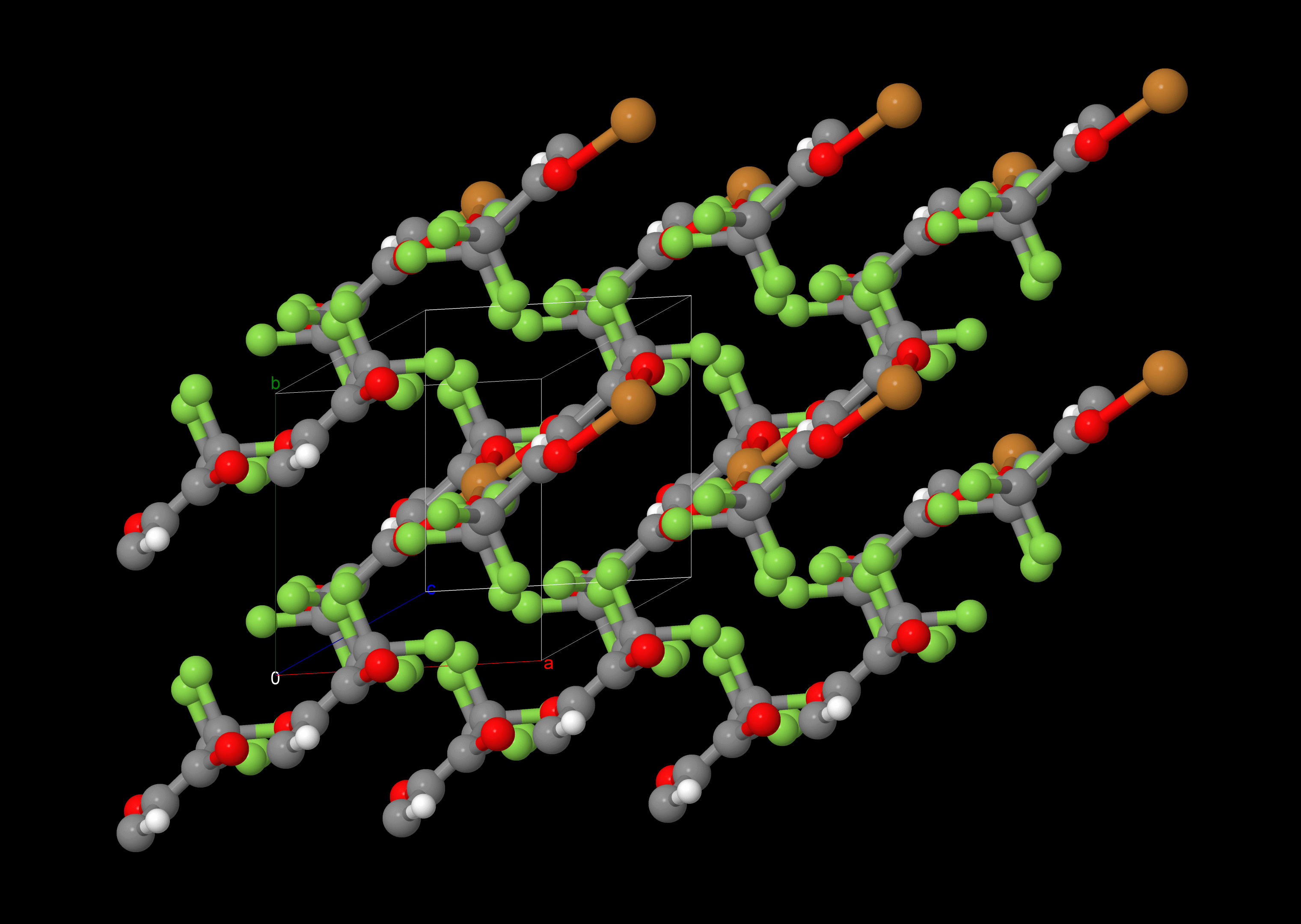

Supplement: Supplementary file 2 [file cg5c00007_si_002.zip › Vibration_Animations/CuAcAcF/67.59.gif]

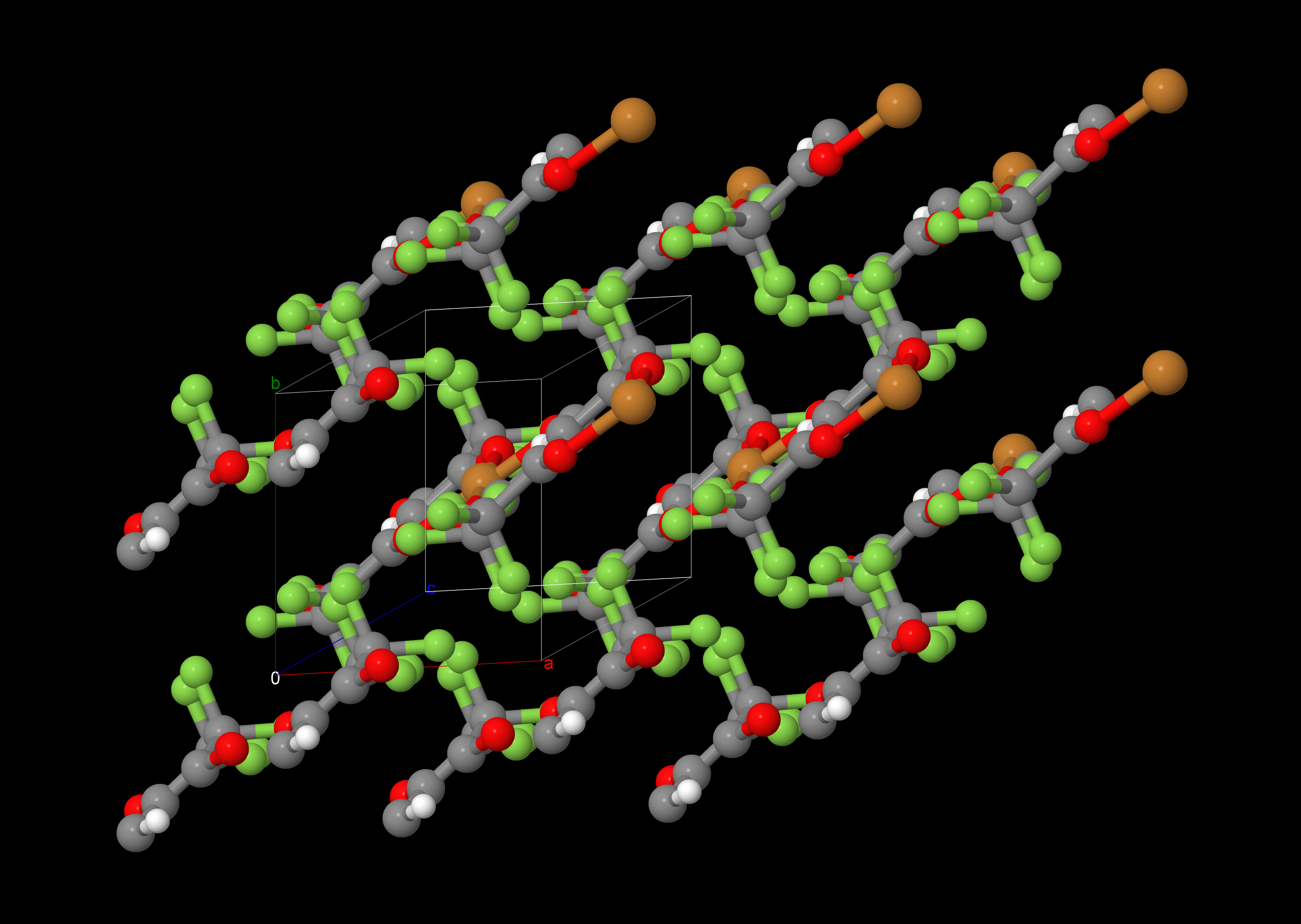

Supplement: Supplementary file 2 [file cg5c00007_si_002.zip › Vibration_Animations/CuAcAcF/62.86.gif]

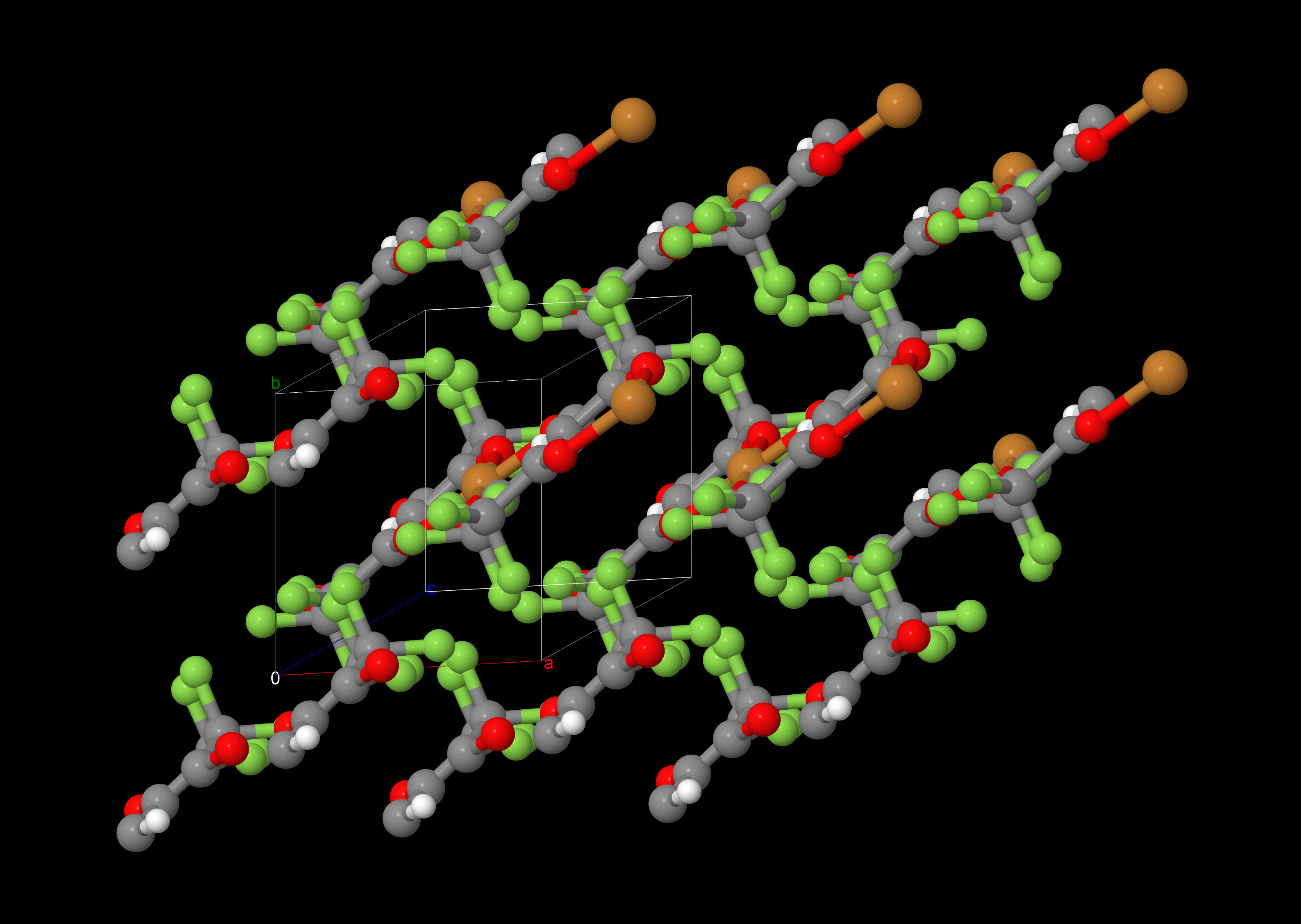

Supplement: Supplementary file 2 [file cg5c00007_si_002.zip › Vibration_Animations/CuAcAcF/129.01.gif]

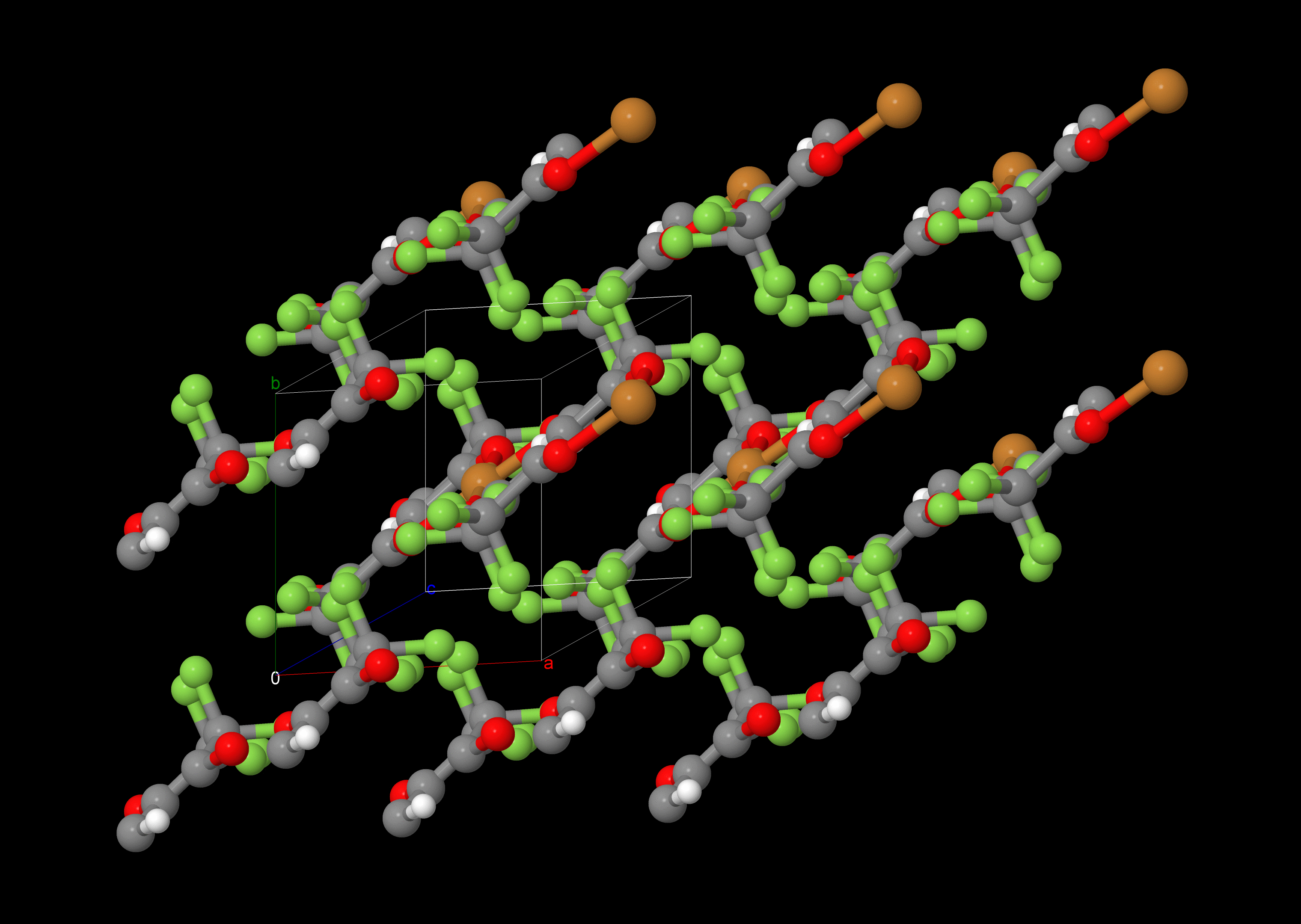

Supplement: Supplementary file 2 [file cg5c00007_si_002.zip › Vibration_Animations/CuAcAcF/121.48.gif]

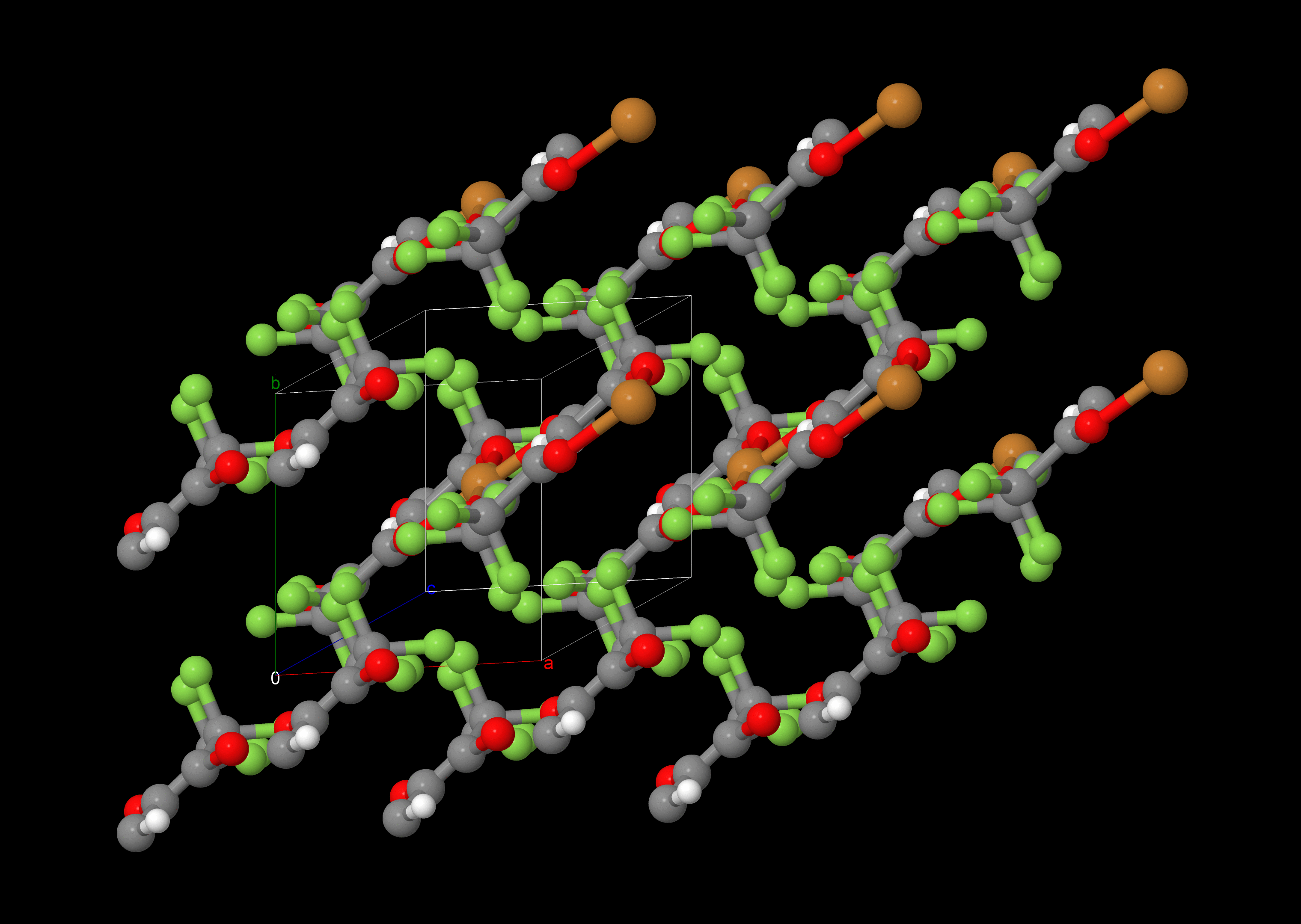

Supplement: Supplementary file 2 [file cg5c00007_si_002.zip › Vibration_Animations/CuAcAcF/122.54.gif]

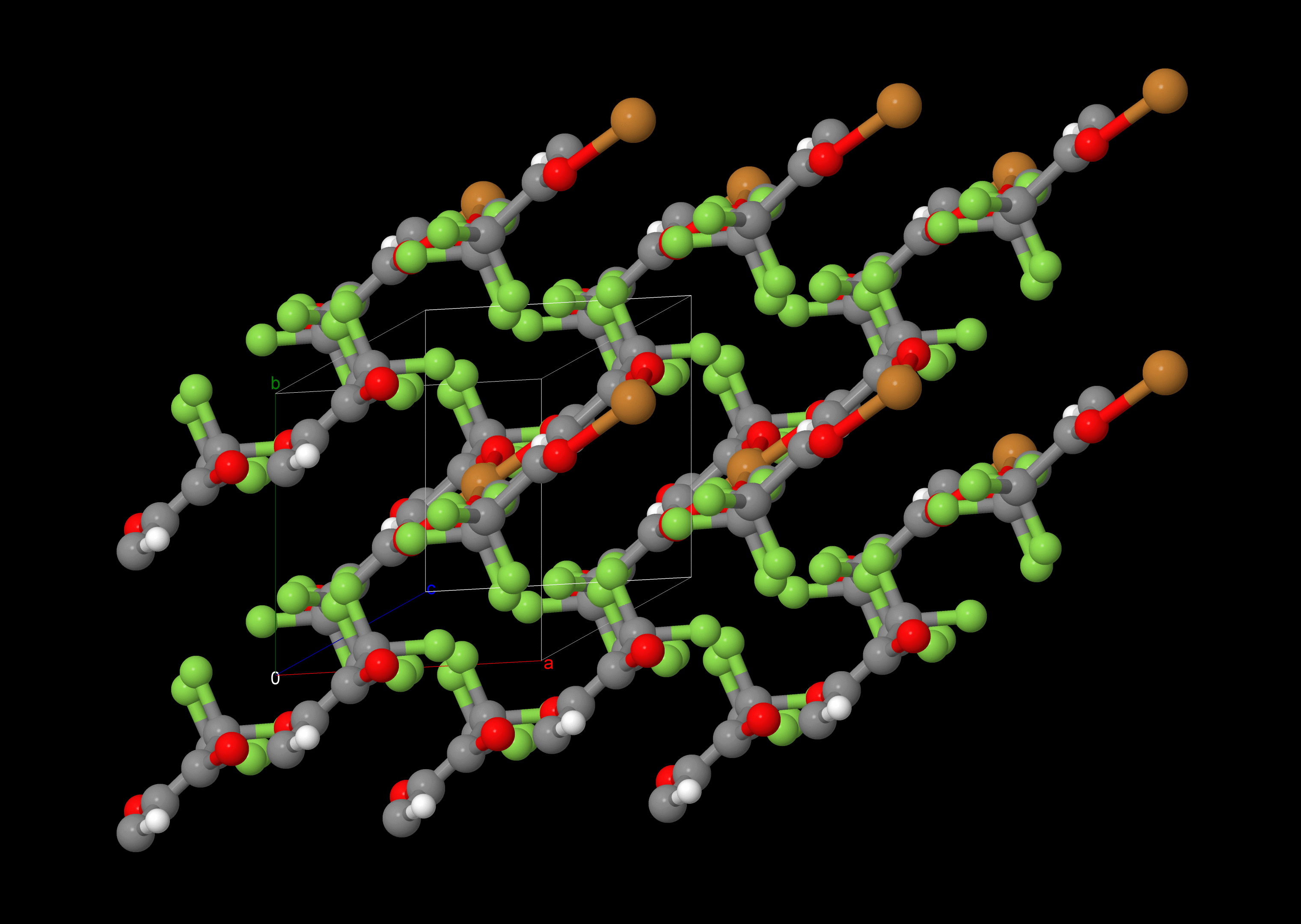

Supplement: Supplementary file 2 [file cg5c00007_si_002.zip › Vibration_Animations/CuAcAcF/141.81.gif]

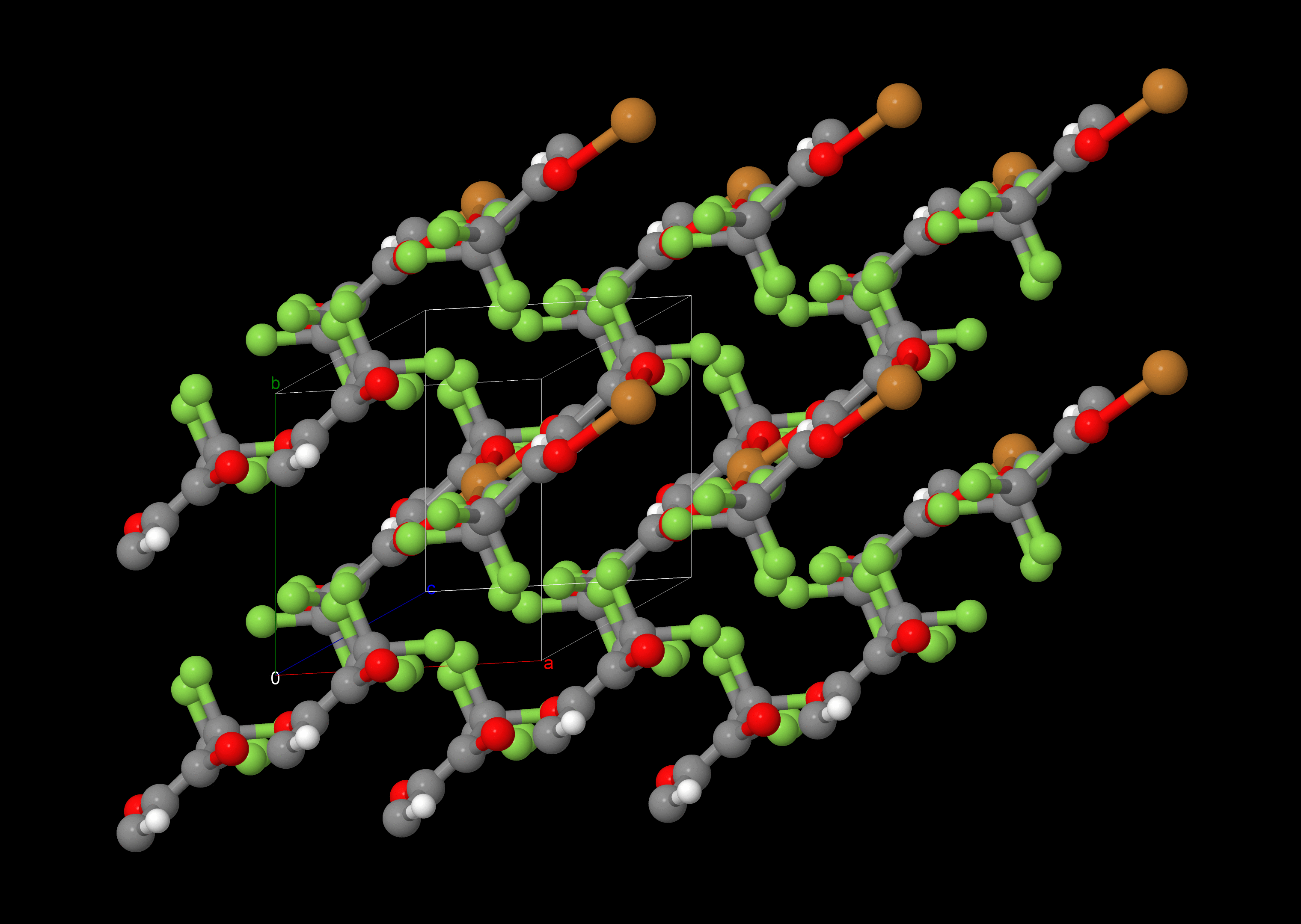

Supplement: Supplementary file 2 [file cg5c00007_si_002.zip › Vibration_Animations/CuAcAcF/40.59.gif]
